# Differentially expressed microRNAs between cattleyak and yak testis

**DOI:** 10.1038/s41598-017-18607-0

**Published:** 2018-01-12

**Authors:** Chuanfei Xu, Shixin Wu, Wangsheng Zhao, TserangDonko Mipam, Jingbo Liu, Wenjing Liu, Chuanping Yi, Mujahid ali Shah, Shumin Yu, Xin Cai

**Affiliations:** 1 School of Life Science and Engineering, Southwest University of Science and Technology, Mianyang, 621010 Sichuan China; 2 College of Life Science and Technology, Southwest University for Nationalities, Chengdu, 610041 Sichuan China; 3 College of Veterinary Medicine, Sichuan Agricultural University, Chengdu, 611130 Sichuan China

## Abstract

Cattleyak are interspecific hybrids between cattle and yak, exhibiting the same prominent adaptability as yak and much higher performances than yak. However, male infertility of cattleyak resulted from spermatogenic arrest has greatly restricted their effective utilization in yak breeding. In past decades, much work has been done to investigate the mechanisms of spermatogenic arrest, but little is known about the differences of the post-transcriptional regulators between cattleyak and yak, which may contribute to the impaired spermatogenesis. MiRNAs, a class of endogenous non-coding small RNA, were revealed to play crucial roles in regulating gene expression at post-transcriptional level. In the present study, we identified 50 differentially expressed (DE) known miRNAs and 11 novel miRNAs by using Illumina HISeq and bioinformatic analysis. A total of 50 putative target sites for the 13 DE known miRNAs and 30 for the 6 DE novel miRNAs were identified, respectively. GO and KEGG analyses were performed to reveal the functions of target genes for DE miRNAs. In addition, RT-qPCR was performed to validate the expression of the DE miRNAs and its targets. The identification of these miRNAs may provide valuable information for a better understanding of spermatogenic arrest in cattleyak.

## Introduction

Cattleyak are interspecific hybrids between cattle (♂) and yak (♀), which exhibit the same prominent adaptability to harsh environment on Qinghai-Tibetan Plateau as yak. Moreover, cattleyak display much higher performances than yak in such economic traits as meat and milk production, and the hybrids have played important roles in promoting the social and economic development in the plateau regions. However, the F1 males of cattleyak are infertile due to spermatogenic arrest and numerous valuable genes cannot be fixed and passed down to next generations^1^. Therefore, spermatogenic arrest of cattleyak has greatly restricted the effective utilization of the hybrids for decades of years. To investigate the mechanisms of spermatogenic arrest of cattleyak, we have compared the testis transcriptomes and proteomes between cattleyak and yak, and identified 2960 DE genes and 645 DE proteins between cattleyak and yak, respectively^2–4^. The significant divergences between DE genes and proteins demonstrated that the expression of a large number of genes may have been inhibited or silenced during post-transcriptional process. MiRNAs are a class of endogenous non-coding small RNA molecules with ~22 nucleotides in length, regulating gene expression at a post-transcriptional level by binding to the 3′-untranslated regions (3′-UTR) of target mRNAs^5–7^. Since the first identified miRNA *lin 4* in *Caenorhabditis elegans*^8,9^, hundreds of miRNAs have been discovered in mammalian species and accumulating evidences show that miRNAs have been involved in diverse biological processes, such as cell proliferation^10^, differentiation^11^ and apoptosis^12^. In addition, many miRNAs are evolutionarily conserved across species^13^, but several miRNAs are expressed in tissue-specific patterns^14^.

Spermatogenesis is a highly-regulated process comprising three main continuous stages of germ cell differentiation: mitotic proliferation of spermatogonia, meiosis of spermatocytes and spermiogenesis of haploid spermatids. In mammals, the differentiation of germ cells to sperms is implicated in the sequential gene expression regulated by extrinsic and intrinsic factors^15^. Dysregulation at any stage of spermatogenesis may cause infertility^16–18^. Several studies have revealed that miRNAs play important roles in regulating the distinct process in mammalian spermatogenesis. MiR-20 and miR-106a were identified to promote the renewal of mouse spermatogonial stem cells (SSCs) at the post-transcriptional level via targeting *STAT3* and *Ccnd1*^19^, and miR-221/222 were found to play a crucial role in maintaining the undifferentiated state of spermatogonia through repression of *KIT* expression^20^. In addition, miR-469 were reported to repress the expression of *TP2* and *Prm2* at the translational level with minor effect on mRNA degradation, which is essential in pachytene spermatocytes for their timely transition to spermiogenesis at later times of spermiogenesis^21^. MiR-122 was shown to regulate chromatin remodeling during sperm development by suppressing the expression of *Tnp2*^22,23^. MiR-355 and miR-181b/c were all up-regulated in the adult testis by targeting *Rsbn1*, a novel homeobox-like protein gene involved in the transcriptional regulation in haploid germ cells^24,25^. As the emerging roles of miRNAs discovered in regulating gene expression at the post-transcriptional level, investigation of the regulatory functions of miRNAs involved in spermatogenesis will contribute to a better understanding of the mechanisms of spermatogenic arrest of cattleyak. In this study, we therefore performed small RNA sequencing and compared the miRNA expression profiles between cattleyak and yak testis. For the first time, we identified the DE miRNAs between the testis samples of cattleyak and yak, some of which were involved in gene regulation during mitotic proliferation, meiosis and spermiogenesis processes. The further characterization of these miRNAs is helpful to reveal the mechanisms of spermatogenic arrest of cattleyak and the identified miRNAs together with their target genes may serve as effective molecular markers in resolving the problems of male infertility of cattleyak in the future.

## Results

### Deep sequencing data of testis small RNAs for cattleyak and yak

**Figure 1:**
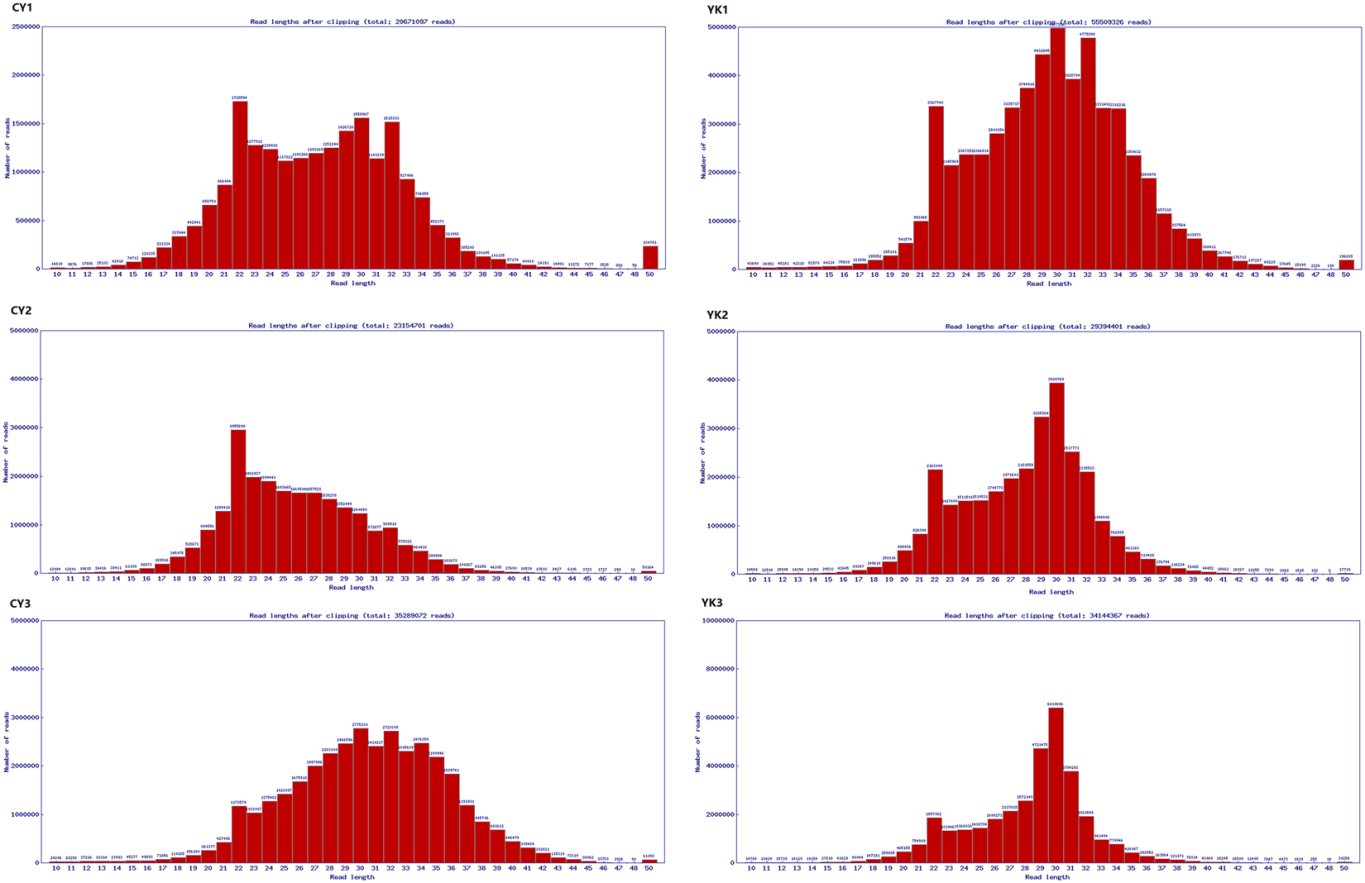
The number of reads from cattleyak and yak testis libraries with different length.

**Figure 2:**
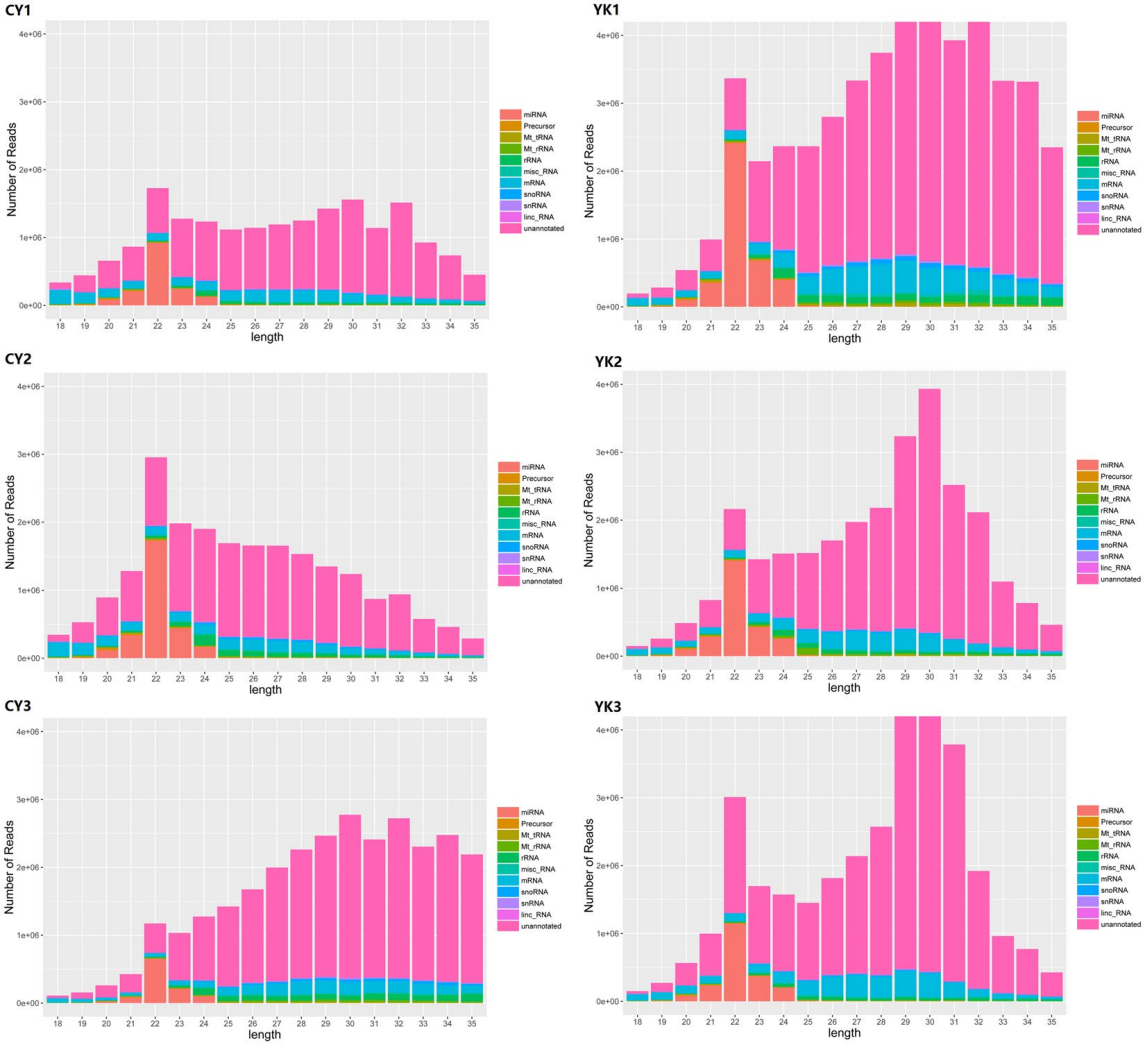
The classification of small RNAs for cattleyak and yak testis miRNA-seq.

In order to identify DE miRNAs involved in spermatogenic arrest of cattleyak, small RNA libraries were constructed from testis total RNAs of cattleyak and yak individuals for Illumina HISeq and miRNA screening. After removing the low quality reads and adaptors, the overall length distribution of clean reads was shown schematically in Fig. 1. All the reads identified from each library ranged from 10 to 50 nt, with two distinctly distributional peaks approximating to 20 and 30 nt. Illumina HISeq provided a total of 20671097, 23154701 and 35289072 reads from the library of CY1 (CY, cattleyak), CY2, and CY3, respectively; while a total of 55509326, 29394401 and 34144367 reads from the library of YK1 (YK, yak), YK2 and YK3, respectively (Fig. 1). After matched to the latest Sanger miRBase and other non-coding RNA database like ncRNA, piRNA and Rfam database using software CLC genomics_workbench 5.5, the clean reads derived from the six samples ranged from 20560465 to 55291115, with the rate of annotated reads ranging from 3.3% to 12.8% and the ambiguously annotated reads ranging from 0.5% to 2.6% (Table 1). The numbers of small RNAs obtained from the six samples ranged from 4920921 to 11294549, with the annotated percentages ranging from 0.2% to 0.8% and ambiguously annotated percentages ranging from 0.1% to 0.2% (Table 1). Subsequently, the annotated small RNAs varying from 18 to 35 nt were classified into several RNA categories such as the known miRNAs, precursors, tRNAs, rRNAs, misc-RNAs, mRNAs, snoRNAs/snRNAs, lincRNAs and unannotated RNAs, in which unannotated RNAs accounted for most of the fractions with the amount ranging from 14157462 to 37310411 and the amount of miRNAs (ranging 20 to 24 nt) varied from 1076993 to 3975469 (Fig. 2, Supplementary Table S1).

**Table 1:** Small RNA and read statistics for cattleyak and yak testis miRNA-seq data.

Sample*	Small RNAs	Annotated Small RNAs (%)	Ambiguously Annotated Small RNAs (%)	Reads	Annotated Reads (%)	Ambiguously Annotated Reads (%)
CY1	5,547,171	30,984 (0.6%)	7,230 (0.1%)	20,560,465	1,689,436 (8.2%)	316,668 (1.5%)
CY2	4,920,921	39,552 (0.8%)	9,916 (0.2%)	23,045,374	2,947,872 (12.8%)	591,505 (2.6%)
CY3	5,960,531	14,873 (0.2%)	4,197 (0.1%)	35,133,851	1,155,575 (3.3%)	183,538 (0.5%)
YK1	11,294,549	26,221 (0.2%)	6,158 (0.1%)	55,291,115	4,126,562 (7.5%)	539,522 (1.0%)
YK2	5,942,346	20,430 (0.3%)	4,833 (0.1%)	29,316,993	2,603,721 (8.9%)	406,772 (1.4%)
YK3	6,813,770	19,200 (0.3%)	4,493 (0.1%)	34,067,132	2,134,608 (6.3%)	329,255 (1.0%)

### Novel miRNAs identified from testis of cattleyak and yak

**Figure 3:**
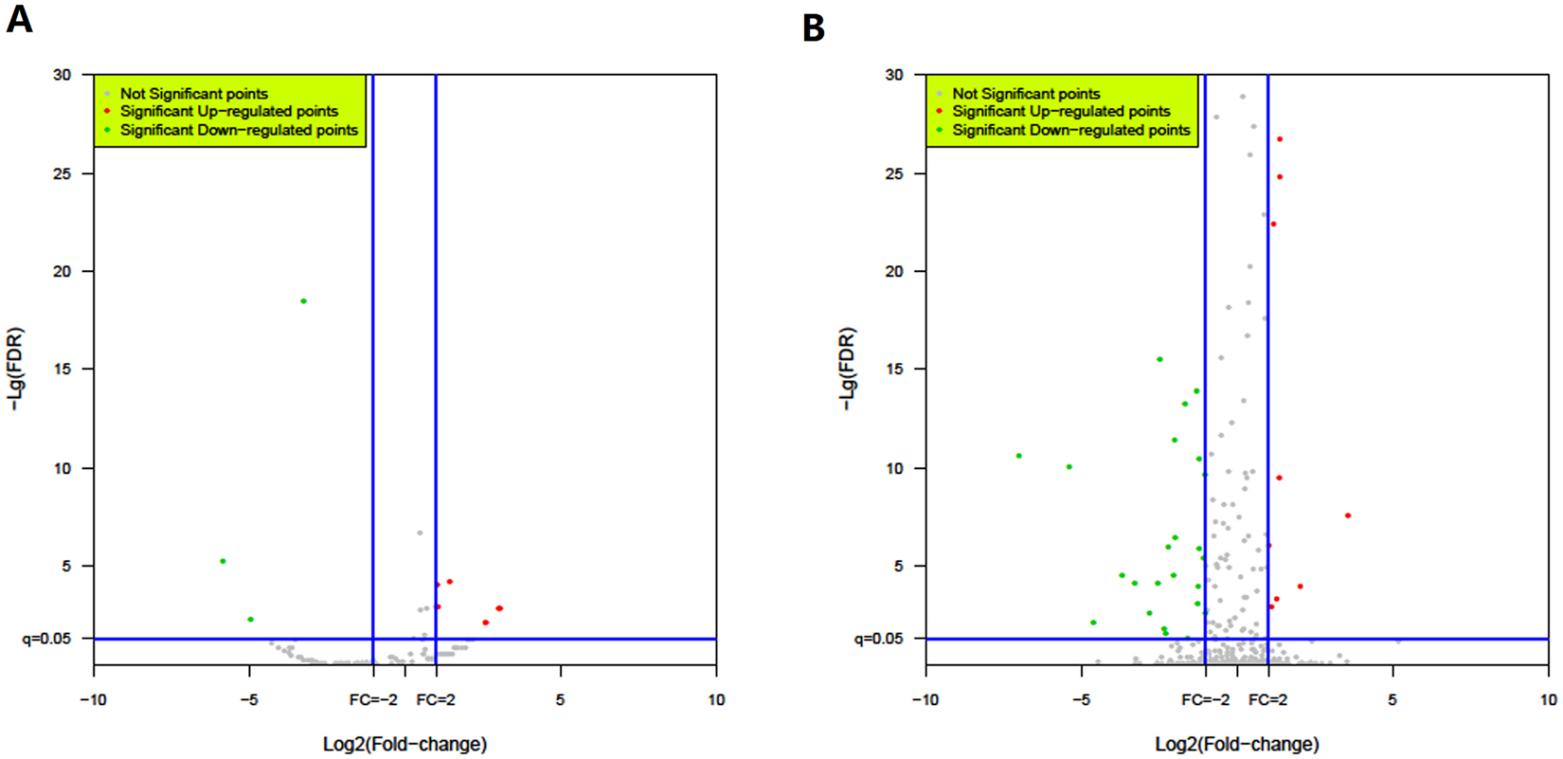
Volcano plot: Log_2_ values of relative expression of known (**A**) and novel (**B**) testis miRNAs (cattleyak related to yak) versus −Log_10_ of false discovery rate (FDR). Horizontal line is at P = 0.05 and vertical lines are at fold change (FC) = ±2.

The special hairpin-like structure of miRNA precursor can be used to predict novel miRNAs. Identification of novel miRNAs by software sRNA Toolkit was based on its secondary structure, the Dicer enzyme cleavage site and the minimum free energy index (MFEI) of the unannotated reads (Table 1) that could be mapped to bovine genome. A total of 168 novel miRNAs matching to 615 hits in the bovine genome were identified from the unannotated reads with the MFEI varying from −161.81 to −13.2 and the novel miRNAs were named according to the species and categories they belonged to. The length of novel miRNAs ranged from 18 to 23 nt, with a distribution peak at 22 nt. Of the 168 novel miRNAs, 88 were identified from cattleyak and 120 from yak libraries, and 40 miRNAs were identified from both cattleyak and yak libraries (Supplementary Table S2). Furthermore, 11 novel miRNAs were identified to be differentially expressed between cattleyak and yak groups, in which 8 miRNAs were significantly upregulated and 3 were downregulated (Fig. 3A, Table 2).

**Table 2:** Summary of DE novel miRNAs in testis from cattleyak (CY) versus yak (YK).

MiRNA name	description	CY_Tpm	YK_Tpm	Fold_change (CY/YK)	p-value	FDR	UP/DOWN regulate	Number of genomic targets
bta-novel-miR-13	ACCTCCCGTGGAGCAGAAGGGCA	21.90231	10.62615	2.061171	3.46E-06	9.83E-05	UP	3413
bta-novel-miR-19	ACTTTTGCCCCTAGTAACGGACT	6.102984	1	6.102984	0.000796	0.008075	UP	144
bta-novel-miR-27	ATCTGTAGTCTCGGCGTCGCACT	8.335362	1	8.335362	7.56E-05	0.001535	UP	871
bta-novel-miR-33	CACCTAGCACTCGCTCGCACC	14.32744	6.812611	2.103076	9.39E-05	0.001213	UP	125
bta-novel-miR-65	GATATTGACATCTCTGGACCC	8.224455	1	8.224455	7.56E-05	0.001535	UP	40
bta-novel-miR-77	GGGCATACTTGTAGACCTTGCC	19.83946	7.238399	2.740862	1.86E-06	6.61E-05	UP	424
bta-novel-miR-118	TCTTAAGATTTGGTGCAATATG	6.0206	1	6.0206	0.000796	0.008075	UP	35
bta-novel-miR-160	TTCTCTTCAGATCGTATAAATC	8.165835	1	8.165835	7.56E-05	0.001535	UP	130
bta-novel-miR-40	CCCGCGAGGGGGCGGGGC	1	57.18054	0.017488	1.29E-07	6.09E-06	DOWN	994
bta-novel-miR-96	TACTGTGCCTTGAATGGG	1	30.7545	0.032516	0.000501	0.005469	DOWN	363
bta-novel-miR-128	TGATTGGTACTTCTTAGAGTGGA	34.14789	325.4347	0.10493	2.39E-21	3.39E-19	DOWN	122

The statistical standards of miRNAs were >2 or <−2 fold change in related groups (CY/YK) and were P < 0.05 (Fisher’s exact test).

### DE miRNAs between testis of cattleyak and yak

To gain insight into the possible roles of miRNAs involved in spermatogenic arrest of cattleyak, it is crucial to investigate the DE miRNA profiles of cattleyak and yak testis. The software edgeR was used to analyze the expression level of miRNAs obtained from both the cattleyak and yak, and DE miRNAs were screened between cattleyak and yak after the number of reads were normalized to transcripts per million (TPM). Volcano plot indicated the pattern of 50 DE known miRNAs (Fig. 3B) between cattleyak and yak, of which 11 were up-regulated and 39 were down-regulated (Table 3). However, of these DE miRNAs, the top three up-regulated miRNAs were bta-miR-592, bta-miR-1247-5p, bta-miR-2484, with an increased fold-change of 11.94, 4.114338, 3.319338, respectively; the top three down-regulated miRNAs were bta-miR-449a, bta-miR-34c, bta-miR-449b, with a decreased fold-change of 0.001319, 0.006306, 0.007821, respectively.

**Table 3:** Summary of differentially expressed known miRNAs in testis from cattleyak (CY) versus yak (YK).

MiRNA name	CY_Tpm	YK_Tpm	Fold-change log_2_ (CY/YK)	p-value	FDR	Up/Down regulated	Number of genomic targets
bta-miR-1247-5p	38.50574	9.358916	4.114338	2.10E-05	0.000101763	UP	2144
bta-miR-19b	1443.226	653.6905	2.207813	3.80E-71	7.16E-70	UP	86
bta-miR-2332	390.6089	150.3797	2.597485	1.47E-26	1.70E-25	UP	90
bta-miR-2411-3p	415.0229	158.272	2.622213	1.49E-28	1.81E-27	UP	858
bta-miR-2484	436.1548	131.3981	3.319338	2.15E-40	2.96E-39	UP	123
bta-miR-2904	443.8361	195.653	2.268486	3.96E-24	4.38E-23	UP	2963
bta-miR-369-3p	61.74624	25.47694	2.423613	0.000100687	0.00046171	UP	3
bta-miR-455-3p	64.47041	29.88945	2.156962	0.000283982	0.001267956	UP	1440
bta-miR-503-3p	150.1078	73.72338	2.036095	1.50E-07	9.09E-07	UP	195
bta-miR-574	151.2986	58.5845	2.582571	4.62E-11	3.41E-10	UP	1096
bta-miR-592	40.52381	3.391165	11.94982	4.01E-09	2.76E-08	UP	32
bta-miR-105a	2.976893	15.04598	0.197853	0.004449884	0.017558069	DOWN	443
bta-miR-10a	3470.423	7410.491	0.468312	1.02E-306	5.76E-305	DOWN	520
bta-miR-126-3p	1074.771	2427.455	0.442756	6.12E-114	1.56E-112	DOWN	35
bta-miR-126-5p	3907.057	7828.106	0.499106	6.83E-280	2.90E-278	DOWN	3
bta-miR-128	107.0503	262.7056	0.407491	1.26E-15	1.19E-14	DOWN	1349
bta-miR-130b	34.10613	80.56888	0.423316	2.51E-05	0.000119293	DOWN	274
bta-miR-133a	29.28078	116.9537	0.250362	4.82E-13	4.15E-12	DOWN	795
bta-miR-135a	54.15177	171.6531	0.315472	6.16E-15	5.60E-14	DOWN	303
bta-miR-139	24.77737	59.1521	0.418875	0.000194892	0.000877875	DOWN	418
bta-miR-143	34501.89	72492.95	0.475934	0	0	DOWN	697
bta-miR-146a	384.0079	1180.822	0.325204	2.41E-91	5.11E-90	DOWN	571
bta-miR-146b	9652.898	40429.24	0.23876	0	0	DOWN	727
bta-miR-147	1.883053	24.12422	0.078057	5.75E-06	2.93E-05	DOWN	647
bta-miR-15b	70.6145	282.1996	0.250229	1.82E-30	2.38E-29	DOWN	1677
bta-miR-16a	710.3616	1479.466	0.480147	3.90E-59	6.85E-58	DOWN	1534
bta-miR-16b	1600.679	4393.994	0.364288	7.94E-287	4.04E-285	DOWN	1138
bta-miR-184	0.508842	12.3765	0.041114	0.001839606	0.007675078	DOWN	811
bta-miR-18a	17.02364	67.22797	0.253223	5.76E-08	3.57E-07	DOWN	338
bta-miR-196a	6.022989	34.96246	0.17227	1.55E-05	7.66E-05	DOWN	607
bta-miR-2318	2.654966	25.76386	0.10305	1.55E-05	7.59E-05	DOWN	29
bta-miR-2419-5p	38.44689	76.92026	0.499828	0.000561739	0.002423095	DOWN	93
bta-miR-2435	2.994194	14.59649	0.205131	0.007573783	0.028769072	DOWN	76
bta-miR-2483-5p	6.7603	20.11475	0.336087	0.012625225	0.046906859	DOWN	194
bta-miR-296-3p	64.49805	136.2474	0.473389	6.70E-07	3.83E-06	DOWN	1881
bta-miR-34a	11.52679	53.05775	0.21725	1.80E-07	1.08E-06	DOWN	2710
bta-miR-34b	8.837222	948.8895	0.009313	1.68E-264	6.12E-263	DOWN	917
bta-miR-34c	108.1892	17155.33	0.006306	0	0	DOWN	1465
bta-miR-375	8.70221	354.1817	0.02457	1.48E-91	3.28E-90	DOWN	351
bta-miR-378	130.7823	265.7867	0.492057	3.23E-11	2.45E-10	DOWN	1821
bta-miR-449a	6.649543	5039.895	0.001319	0	0	DOWN	2360
bta-miR-449b	0.339228	43.3717	0.007821	2.78E-12	2.32E-11	DOWN	2342
bta-miR-449c	1	41.84592	0.023897	1.06E-11	8.55E-11	DOWN	1925
bta-miR-451	93.67503	217.4898	0.43071	4.00E-12	3.28E-11	DOWN	18
bta-miR-484	54.0478	125.1392	0.431901	2.11E-07	1.25E-06	DOWN	3369
bta-miR-486	1142.422	3311.228	0.345015	3.26E-234	1.11E-232	DOWN	847
bta-miR-6123	3.087651	21.62779	0.142763	0.000539356	0.00234643	DOWN	697
bta-miR-6526	1168.153	12648.09	0.092358	0	0	DOWN	2092
bta-miR-767	10.86563	44.6083	0.243579	5.59E-06	2.88E-05	DOWN	1521
bta-miR-9-5p	20.94206	116.7742	0.179338	3.17E-17	3.05E-16	DOWN	261

The statistical standards of miRNAs were >2 or <−2 fold change in related groups (CY/YK) and were P < 0.05 (Fisher’s exact test).

### Prediction of target genes for DE miRNAs

**Figure 4:**
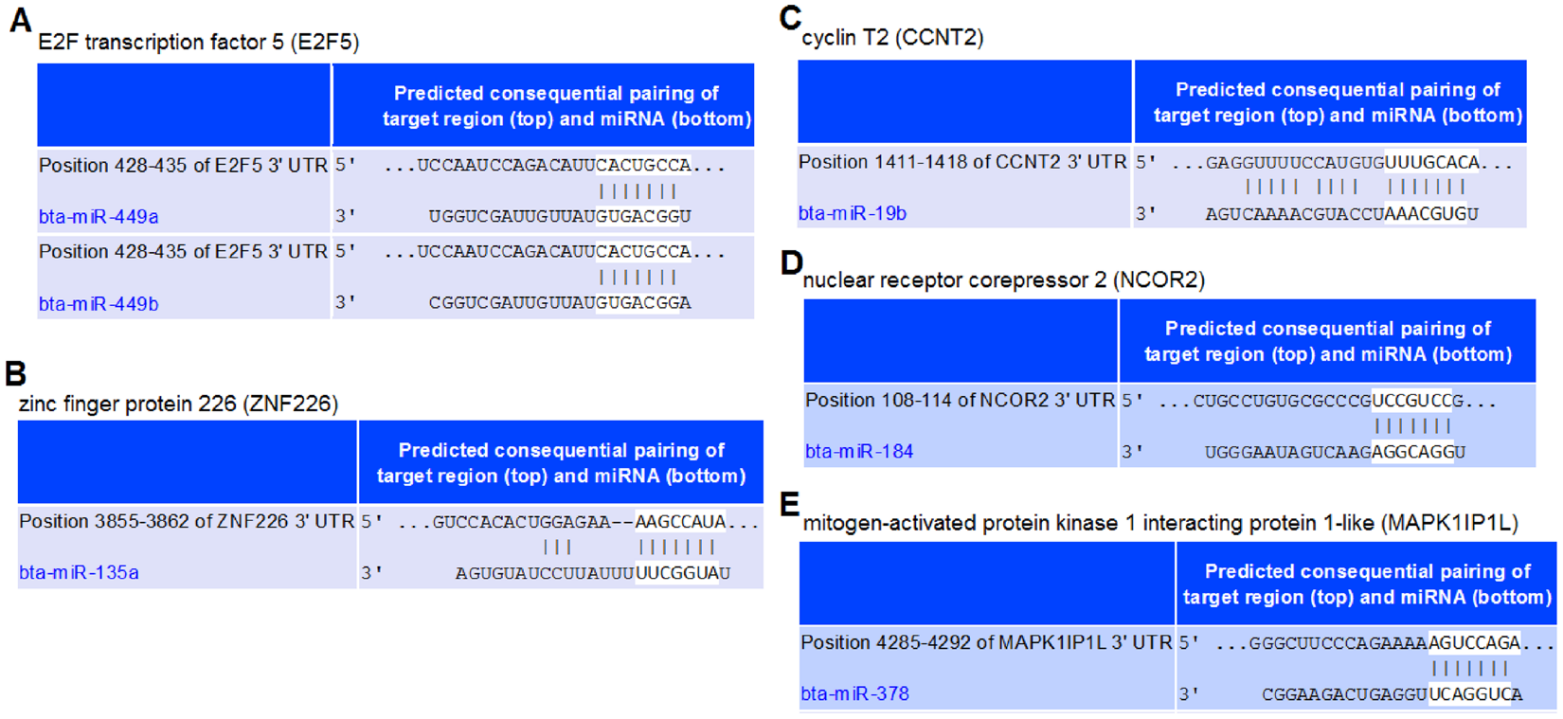
Predicted targets and binding sites of the DE known miRNAs between cattleyak and yak.

To identify the potential function roles of these miRNAs, target gene prediction was performed using software miRanda. The potential targets of the DE miRNAs were screened out with total score ≧172 and total energy ≦−20.00. Target genes for the 13 DE known miRNAs between cattlyak and yak were shown in Supplementary Table S3 and for the 6 DE novel miRNAs were shown in Supplementary Table S4. A total of 50 putative target sites for the 13 DE known miRNAs and 30 for the 6 DE novel miRNAs were identified, respectively. Figure 4 indicated the target sites for 6 DE miRNAs. The results showed that most miRNAs had multiple distinct target genes possessing diverse functions. For instance, a total of 18 putative target genes for bta-miR-135a were identified and 9 putative target genes for bta-novel-miR-160 were identified. The putative target sites for bta-miR-135a were zinc finger family genes, *CSPP1*, *CUBN* and so on.

### GO and KEGG enrichment of the target genes for DE miRNAs

**Figure 5:**
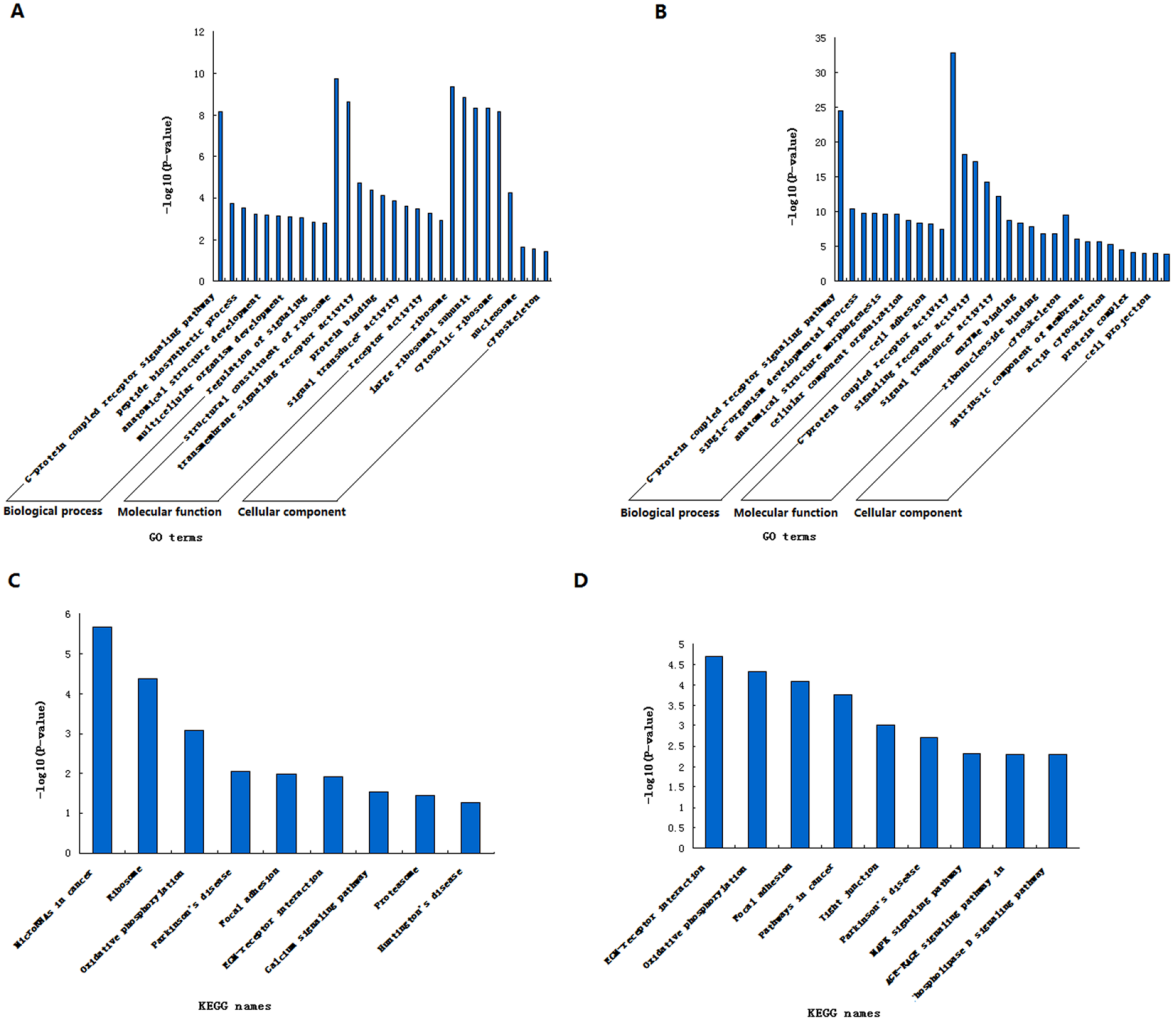
Enrichments of target genes for DE miRNAs. The top 10 items of GO enrichments of target genes for DE known miRNAs (**A**) and for DE novel miRNA (**B**) were based on biological process, molecular function and cellular component, respectively. GO terms in each ontological category were ranked according to decreased −log10 of p values listed on the y-axis. The top 10 pathways of KEGG enrichments of target genes for DE known miRNAs (**C**) and for DE novel miRNAs (**D**) were ranked according to decreased −log10 of p values listed on the y-axis.

To understand the roles of DE miRNAs between cattleyak and yak, a total of 46887 target genes for DE known miRNAs and 181138 for DE novel miRNAs were subjected to GO and KEGG enrichment. As a result, a total of 114 significantly enriched GO terms were obtained for target genes of DE known miRNAs, in which G-protein coupled receptor signaling pathway (p = 6.88E-09), structural constituent of ribosome (p = 1.77E-10) and ribosome (p = 4.32E-10) were the top listed GO terms involved in biological process, molecular function and cellular component, respectively (Fig. 5A, Supplementary Table S5). Meanwhile, 575 GO terms were significantly enriched for target genes of DE novel miRNAs, in which G-protein coupled receptor signaling pathway (p = 2.99E-25), G-protein coupled receptor activity (p = 1.24E-33) and cytoskeleton (p = 3.69E-10) were the top listed GO terms involved in biological process, molecular function and cellular component, respectively (Fig. 5B, Supplementary Table S6). KEGG enrichment results showed that only 8 significantly enriched pathways were obtained for target genes of DE known miRNAs, in which MicroRNAs in cancer (p = 2.20E-06), Ribosome (p = 4.32E-05) and Oxidative phosphorylation (p = 0.000827) were the top listed three pathways (Fig. 5C, Supplementary Table S7). Meanwhile, 53 pathways were significantly enriched for target genes of DE novel miRNAs, in which ECM-receptor interaction (p = 2.00E-05), Oxidative phosphorylation (p = 4.67E-05) and Focal adhesion (p = 8.21E-05) were the top listed three pathways (Fig. 5D, Supplementary Table S8).

Further analysis revealed that hundreds or thousands of target genes were enriched in each GO term or KEGG pathway (Supplementary Tables S5–S8), and these genes were mainly involved in such biological processes as cellular signal transduction, cellular development, cellular differentiation and protein modification. For example, 3024 target genes were involved in developmental process, 3178 in single-organism developmental process, 1551 in intracellular signal transduction, 2059 in cell differentiation and 855 in cell cycle (Supplementary Table S5); 112 genes were involved in MicroRNAs in cancer, 163 in Focal adhesion and 73 in ECM-receptor interaction (Supplementary Table S7).

### RT-qPCR validation of DE miRNAs and their target genes

**Figure 6:**
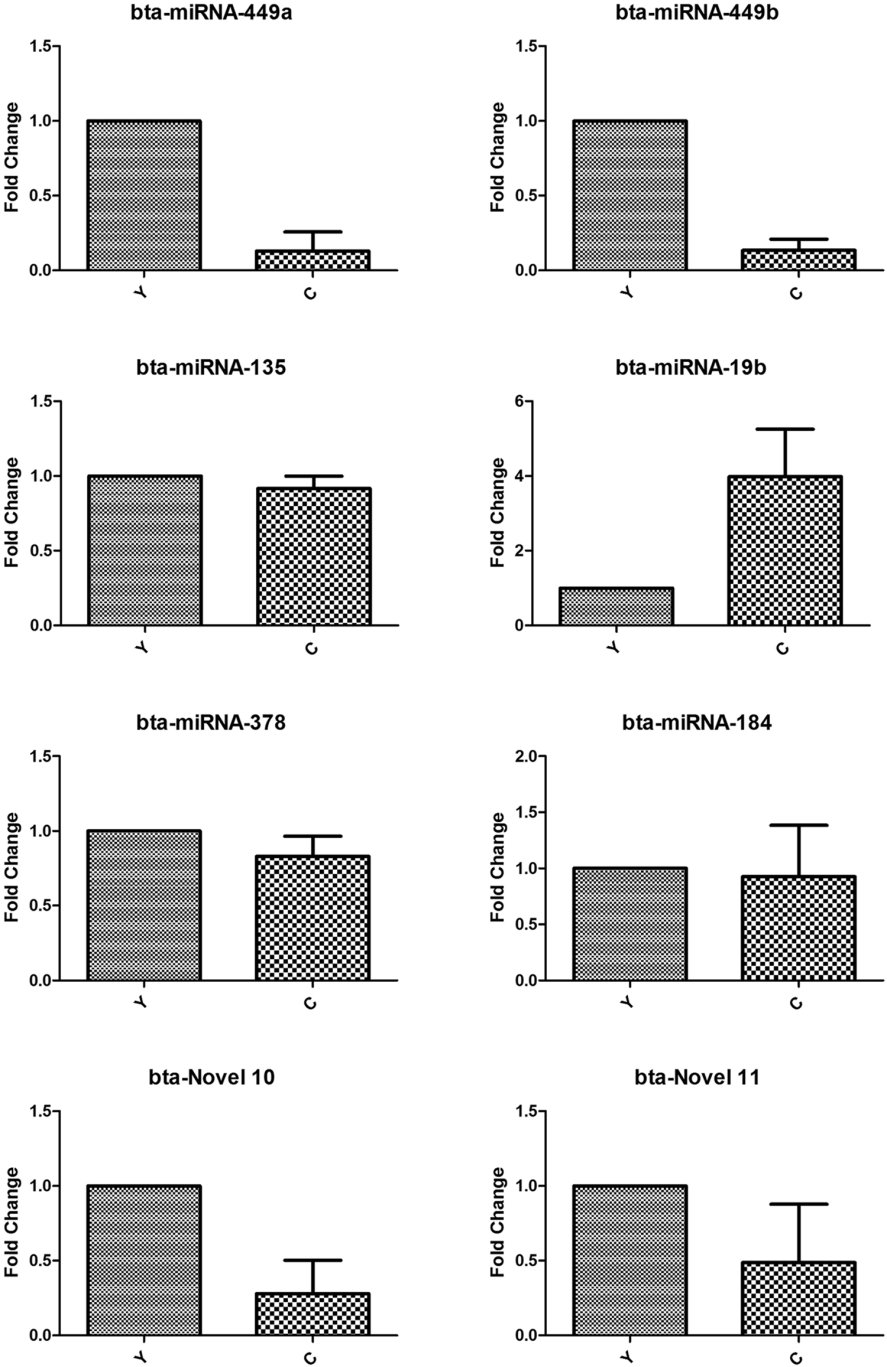
The expression of miRNAs in yak (Y) and cattleyak(C) testis tissues were validated by RT-qPCR.

**Figure 7:**
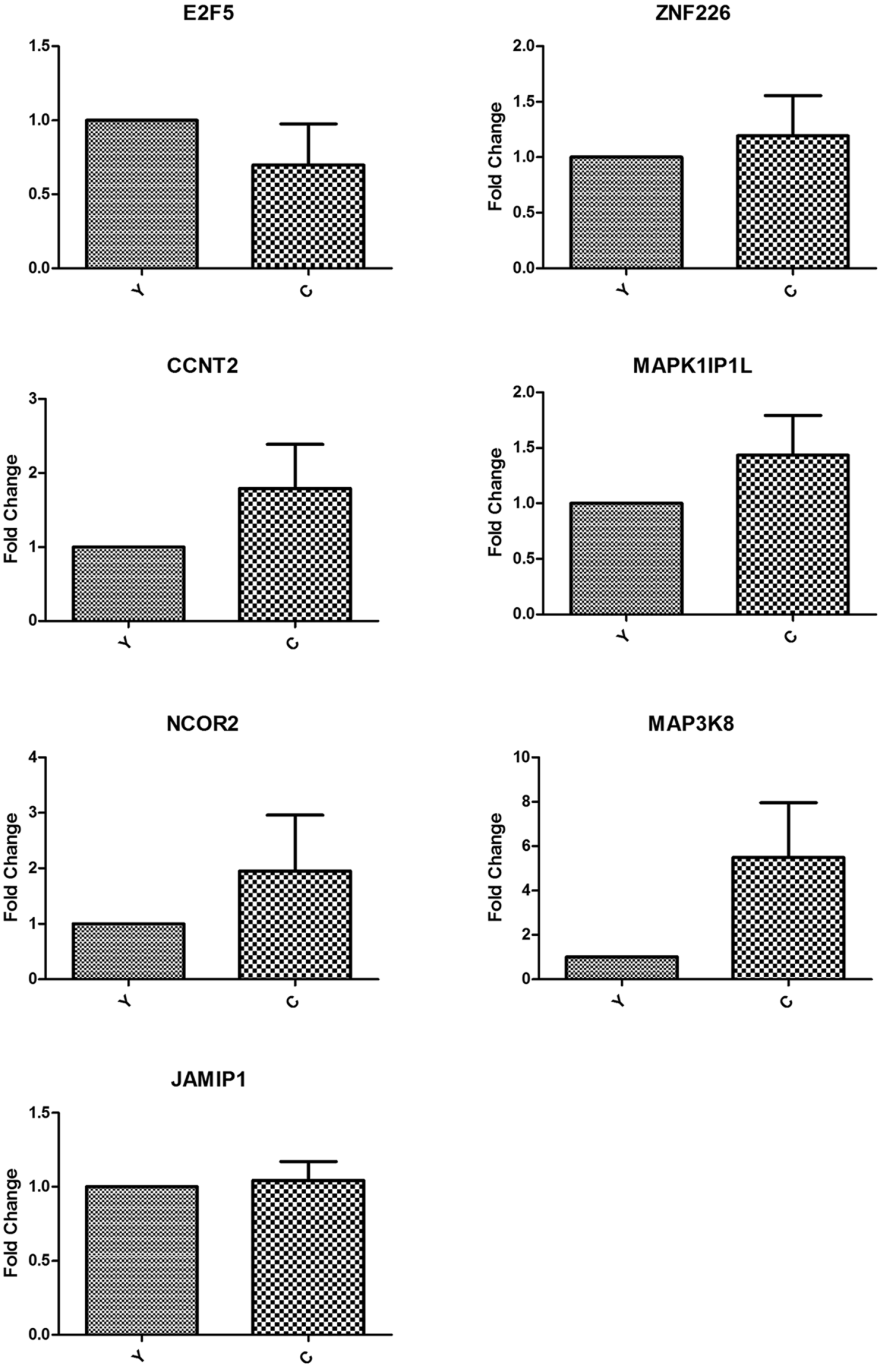
The expression of target genes for miRNAs in yak (Y) and cattleyak (C) testis tissues were detected by RT-qPCR.

To validate the expression level of DE miRNAs, stem-loop RT-qPCR was performed on 6 known miRNAs (bta-miRNA-449a/b, bta-miRNA-135, bta-miRNA-19b, bta-miRNA-378 and bta-miRNA-184) and 2 novel miRNAs (bta-novel 10 and bta-novel 11). *RPS18* was used as the internal reference and the sequences of primers were listed in the Supplementary Table S9. As shown in Fig. 6, comparison of miRNA expression revealed that the expression of all the miRNAs selected were downregulated in cattleyak with respect to yak except the upregulation of bta-miRNA-19b, which was fully consistent with their expression patterns obtained from sequencing data. In addition, 7 target genes (*E2F5*, *ZNF226*, *CCNT2*, *MAPK1IP1L*, *NCOR2*, *MAP3K8* and *JAMIP1*) of these miRNAs were selected to validate their expression levels. *β-actin* was used as the internal reference and the sequences of primers were listed in the Supplementary Table S10. As shown in Fig. 7, the expression levels of all the target genes were upregulated in cattleyak except *E2F5*, which indicated that expression of the target genes were almost inhibited by the corresponding miRNAs. Meanwhile, the expression of *E2F5* and *CCNT2* were not found to be significantly inhibited by bta-miRNA-449a/b and bta-miRNA-19b, respectively.

## Discussion

Since the discovery of miRNAs and their roles in post-transcriptional gene regulation, many studies have shown that they were implicated in the regulation of diverse biological processes^10–12^ and several miRNAs have been found to be involved in multiple processes of spermatogenesis such as mitotic proliferation of spermatogonia, meiosis of spermatocytes and spermiogenesis of haploid spermatids^26–29^. However, no study was conducted to characterize miRNAs involved in spermatogenic arrest of cattleyak. In our study, 50 DE known miRNAs and 11 novel miRNAs were identified between the testis samples of cattleyak and yak by using Illumina HISeq and bioinformatic screening. The majority of miRNAs were 21–23 nt in length, which was consistent with previous reports on the length distribution of miRNA identified in the adipose tissue and mammary gland of bovine^30,31^. Stem-loop RT-qPCR was performed to validate the expression level of known and novel miRNAs, and the results were highly consisted with the sequencing data. The identification of these miRNAs may provide valuable information to a better understanding of the mechanisms of spermatogenic arrest of cattleyak.

In this work, the target genes for DE miRNAs were identified to be involved in such biological processes as cellular signal transduction, cellular development, cellular differentiation and protein modification (Supplementary Tables S5, S6), which was bound to be associated with specific spermatogenic processes in cattleyak. During spermatogenesis, the regulation of balance between maintenance of undifferentiated SSCs and transition to a differentiating state could ensure adequate numbers of spermatogonia undergoing spermatogenesis and lifelong supply of spermatozoa. The downregulation of miR-135a was revealed to activate *FoxO1* gene involved in cellular proliferation, which resulted in the decreased number of SSCs and failure of spermatogonial stem cell maintenance in cryptorchid testes^32^. The expression of miR-378 was also significantly downregulated in prostate cancer tissues, which was identified to suppress cell growth through downregulation of *MAPK1* gene involved in cell proliferation process^33^. Meanwhile, miR-184 was reported to downregulate *Ncor2* gene and result in significantly lower number of cells in the G1 phase in mouse spermatogenesis^34^. In the present study, the downregulation of bta-miR-135a, bta-miR-378 and bta-miR-184 could contribute to the decreased number of SSCs and spermatogenic alterations in cattleyak, which was consistent with the cellular distribution characteristics in cattleyak testis revealed by histological analysis^1–3^. On the other hand, the over expression of miR-19b-3p was reported to repress the expression of *USP32, RAB18* and *Dusp6* involved in cell differentiation process and contribute to the inhibited proliferation and slow-downed cycle of SH-SY5Y cells^35^. Over expression of miR-592 could promote the cell proliferation by downregulating the expression of *FOXO3* involved in cell cycle process and promote the proliferation of prostate cancer cells^36^. Therefore, inhibited expression of *USP32, RAB18* and *Dusp6* caused by bta-miR-19b-3p may be conducive to the maintaining of undifferentiated SSCs in cattleyak, while downregulation of *FOXO3* by bta*-*miR-592 could contribute to the transition of SSCs to a differentiating state, which was consistent with the conclusions from our previous study that spermatogenic arrest of cattleyak started at the stage of spermatogonial differentiation and get aggravated during meiosis^3^.

Meiosis and spermiogenesis, the two successive phases of spermatogenesis, must be tightly regulated and any mistake in these processes will lead to spermatogenic arrest. As known, the progression of these processes implicates complex gene expression regulation at post-transcriptional levels^15–18^. To date, few miRNAs have been shown to play critical roles in these processes. Upon the initiation of meiosis, the miR-449 cluster and miR-34b/c functioned redundantly in down-regulating the activities of the E2F-pRb pathway, which allowed male germ cells to exit from the mitotic cycle and to enter the meiotic program in murine testes^37^. MiRNA-34c was mainly expressed in the late stages of meiosis and was likely to influence germ cell fate during this period via downregulation of *TGIF2* and *NOTCH2*^38^. Over expression of miR-34c was revealed to promote the expression of *Nanos3*, *Scp3*, and *Stra8* genes involved in meiosis in mouse spermatogenesis^39^. As the essential roles played by these miRNAs in meiosis, the downregulation of bta-miR-34b/c and bta-miR-449 cluster in cattleyak could repress the expression of *Nanos3*, *Scp3*, and *Stra8* and contribute to the failure of the transition from mitosis to meiosis and the subsequent spermiogensis, which was consistent with our previous finding that spermatogenic arrest of cattleyak got aggravated during meiosis^3^. Although the spermatogenic arrest occurred during spermatogonial differentiation, the balance between undifferentiated SSCs and differentiated spermatogonic cells should be partially maintained in cattleyak.

In conclusion, for the first time we provided a useful resource for further elucidation of the regulatory role of potential miRNAs in spermatogenic arrest of cattleyak. Using Illumina HISeq and bioinformatic analysis, 50 DE miRNAs and 11 novel miRNAs were identified between yak and cattleyak. The GO and KEGG enrichment for the predicted target genes indicated the functional complexity of miRNAs. The downregulation of bta-miR-135a, bta-miR-378 and bta-miR-184 could contribute to the decreased number of SSCs and spermatogenic alteration in cattleyak. Furthermore, the downregulation of bta-miR-34b/c and bta-miR-449 cluster may have caused the transition failure from mitosis to meiosis and the subsequent spermiogensis in cattleyak. In the future, much effort should be exerted to investigate the role of individual miRNA involved in spermatogenic arrest of cattleyak and explore the pathways involved to reveal the mechanisms of cattleyak infertility.

## Materials and Methods

### Animals and testis processing

Three cattleyak (named C1, C2 and C3) and three yak (named Y1, Y2 and Y3) aged 12 months were sampled from a Maiwa yak population fed on a pasture in Hongyuan county, Sichuan province of China, in which cattleyak were F1 generation of Simmental and Maiwa yak. Testis of each animal was obtained by veterinary surgical operation and the caudal epididymis was firstly resected. Then fat and fascia surrounding the testis were resected and two crosscut slices of the testicular tissues in the middle of testis were obtained by fine scale dissection. All crosscut slices were snap frozen in liquid nitrogen (−196 °C), transported to laboratory and stored at −80 °C until RNA isolation.

### Ethics statement

Sample collection was carried out under the license in accordance with the Guideline for Care and Use of Laboratory Animals of China and all protocols were approved by the institution Review Board of Southwest University of Science and Technology.

### RNA preparation, miRNA cloning and sequencing

Small RNAs were extracted from the six frozen testis samples using mirVana™ miRNA Isolation Kit (Cat #. AM1561, Austin TX, USA) according to the manufacturer’s instructions. The integrity and concentration of total RNA were assessed using Agilent 2100 Bioanalyzer (Agilent technologies Santa Clara, USA) to meet the requirement of the Illumina HiSeqTM 2000 sequencing platform. The RNA 3′-adapter was specifically modified to target miRNAs and other small RNAs that have 3′-hydroxyl groups resulted from enzymatic cleavage by Dicer or other RNA processing enzymes. The adapters were ligated to each end of the RNA molecule and a RT reaction was used to create single stranded cDNA. The cDNA is amplified though PCR and followed by gel purification to generate library products. The libraries were then sequenced separately using Illumina HISeq and generated a total of 20732767, 23210984 and 35385105 reads from three testis libraries of cattleyak, and a total of 55707150, 29469263 and 34228475 reads from three testis libraries of yak.

### Statistical analysis

Fastx (Fastx_toolkit-0.0.13.2) was used to remove the low quality reads and adaptors obtained from the six libraries. The clean reads of these libraries were matched to the latest Sanger miRBase (http://www.mirbase.org/) and other noncoding databases like ncRNA (http://asia.ensembl.org/index.html), piRNA (http://www.ncbi.nlm.nih.gov/and http://pirnabank.ibab.ac.in/) and Rfam database (http://rfam.sanger.ac.uk/) with a tolerance of two mismatches using the software of CLC genomics_workbench 5.5. Sequences that did not overlap with any annotated sequence were classified as unannotated reads. Next, the annotated small RNAs varying between 18 to 35 nt were classified into several RNA categories such as the known miRNAs, precursors, tRNAs, rRNAs, misc-RNAs, mRNAs, snoRNAs/snRNAs, lincRNAs and unannotated RNAs. The software sRNA Toolkit was used to identify the novel miRNAs based on its secondary structure, Dicer enzyme cleavage site and minimum free energy indexes (MFEI) of the unannotated reads which could be mapped to bovine genome.

### Differentially expression analysis

To compare the miRNA expression levels between the two samples to determine the DE miRNAs, the software edgeR was utilized to analyze the expression level of miRNAs obtained from the two samples and DE miRNAs were screened between catlleyak and yak after the number of reads were normalized to transcripts per million (Actual miRNA count/Total count of clean reads*1000000, TPM). Fold-change formula: Fold_change = log_2_ (CY_TPM_/YK_TPM_). P-value formula:

1 $$P(X/Y)=(\frac{{N}_{2}}{{N}_{1}}){\frac{(X+Y)}{X!Y!{(1+\frac{{N}_{2}}{{N}_{1}})}^{(X+Y+1)}}}_{D(Y\ge {Y}_{{\rm{\max }}}|X)=\sum _{Y\ge {Y}_{{\rm{\max }}}}^{\infty }p(Y|X)}^{C(Y\le {Y}_{{\rm{\min }}}|X)=\sum _{Y=0}^{Y\le {Y}_{{\rm{\min }}}}p(Y|X)}$$

N_1_ and X represent the total number of clean reads and normalized expression level of a given miRNA in the small RNA library obtained from cattleyak, respectively, and N_2_ and Y represent the total number of clean reads and normalized expression level of a given miRNA in the small RNA library generated from yak, respectively. The significant differential expressed miRNAs were selected according to Fold change and P-value with the criteria: Fold change >2 or <−2 and P-value < 0.05.

### *In silico* analysis of identified miRNAs and target prediction

Target prediction was performed using software miRanda and the rules were used for target prediction in term of the following criteria: targets located in the 3′-UTR region, seed length of at least 7 base pairs (bp), and the minimum free energy (MFE). The potential targets of the DE miRNAs were screened out with total score ≧172 and MFE ≦−20.09.

### GO and KEGG enrichment of the target genes for DE miRNAs

GO enrichment is an effective and efficient tool to classify the target genes for DE miRNAs based on the specific biological functions. KEGG pathways are applied to elucidate *in vivo* comprehensive inferences of reactions such as metabolic pathways or signal transduction pathways. To depict the interaction of the target genes for DE miRNAs involved in the same certain biological functions of spermatogenic arrest of cattleyak, we employed enrichment of GO and KEGG pathway. These two methods calculated the target gene numbers for every term or pathway after comparing to a genome background and then used the hypergeometric test to filter the significantly enriched term or pathway. The same calculating formula was developed to do the analyses described as follows:

2 $$P=1-\sum _{i=0}^{m-1}\frac{(\frac{M}{i})(\frac{N-M}{n-i})}{(\frac{N}{n})}$$

N is the number of all genes with GO or KEGG annotation; n is the number of target genes in N; M is the number of all genes that are annotated to certain GO terms or specific pathways; m is the number of target genes in M. The significantly enriched GO terms or pathways were defined after the calculated p-value went though Bonferroni Correction, taking corrected p-value ≦0.05 as a threshold.

### RT-qPCR validation of DE miRNAs and their target genes

Total RNA was extracted from the six frozen testis samples using TRIzol reagent (Invitrogen, CA, USA) according to the manufacturer’s instructions. The integrity and concentration of total RNA were assessed using theAgilent 2100 Bioanalyzer (Agilent Technologies, Palo Alto, CA, USA). Stem-loop reverse transcription (RT) and real-time polymerase chain reaction (qPCR) with SYBR Green were used for the quantification of miRNA expression. Total RNA (1 µg) was reversely transcribed with PrimeScript^TM^ RT reagent Kitwith gDNA Eraser (TaKaRa, Dalian, China) using a stem-loop RT primer for each tested miRNA. The cDNA was then used for real-time PCR quantification of miRNA using the miRNA specific forward primer and the universal reverse primer. The bovine ribosomal protein S18 (*RPS18*) (GenBank NO. NM_001033614.1) gene was used as an endogenous control. The primers for stem-loop RT and miRNA quantification were listed in Supplementary Table S4. Real-time quantitative PCR was performed in triplicate using a Bio-Rad CFX 96™ Real Time Detection System in a 20 µL reaction comprising 100 ng cDNA for each miRNA, 0.4 µM forward and reverse primers, and 10 µL of 2 × SYBR^®^Premix Ex Taq^TM^ II (TaKaRa, Dalian, China). Reactions were performed at 95 °C for 30 s, followed by 40 cycles of 95 °C for 10 s, 60 °C for 10 s, and 68 °C for 20 s. Individual samples were run in triplicate. In the end of the PCR cycles, melting curve analysis was performed to validate the specific generation of the expected PCR products. The quantification of each miRNA relative to *RPS18* gene was calculated using the2^−ΔΔCt^ method.

Real time PCR was also carried out to validate the expression of the target genes *E2F5*, *ZNF226*, *CCNT2*, *MAPK1IP1L*, *NCOR2*, *MAP3K* and *JAKMIP1* in the testis of cattleyak and yak according to the methods described previously^3^. The primers for the quantification of these genes were listed in Supplementary Table S5. The relative expression level of mRNA for each gene was calculated using *b-actin* as an endogenous reference gene using the 2^−ΔΔCt^ method.

### Statistical analysis

All experiments were performed in triplicate and all data were presented as mean ± SEM. The significantly differentially expressed miRNAs between cattleyak and yak were selected according to Fold change and P-value with the criteria: Fold change >2 or <−2 and P-value < 0.05, using the analysis of variance (ANOVA) and a 2-tailed t-test.

### Availability of data and material

All analyzed data in this study are included in this published article and all RNA-Seq data in this study are available in NCBI under accession number: SRP117203.

## Electronic supplementary material

Supplementary Information

Table S5

Table S6

Table S7

Table S8
