# Supplementary material for: Differentially expressed microRNAs between cattleyak and yak testis
Source: Sci Rep. 2018 Jan 12;8:592. doi: 10.1038/s41598-017-18607-0 (PMC5766512; doi:10.1038/s41598-017-18607-0)
Supplement: Supplementary file 1 — Supplementary Information [file 41598_2017_18607_MOESM1_ESM.pdf]

Differentially expressed microRNAs between cattleyak and yak testis

Chuanfei Xu[1], Shixin Wu[1], Wangsheng Zhao[1], TserangDonko Mipam[2], Jingbo Liu[1], Wenjing Liu[1], Chuanping Yi[1], Mujahid ali Shah[1], Shumin Yu[3] & Xin Cai[1]

[1]School of Life Science and Engineering, Southwest University of Science and Technology, Mianyang 621010, Sichuan, China. [2]College of Life Science and Technology, Southwest University for Nationalities, Chengdu 610041, Sichuan,China. [3]College of Veterinary Medicine, Sichuan Agricultural University, Chengdu 611130, Sichuan, China.

Correspondence and requests for materials should be addressed to X.C. (email: caixin2323@126.com)

Sample	miRNA	Precursor	Mt_tRNA	Mt_rRNA	rRNA	misc_RNA	mRNA	snoRNA	snRNA	linc_RNA	unannotated
CY1	1,598,540	87,072	84,933	96,857	430,289	117,125	2,095,578	236,687	107,022	0	14,157,462
CY2	2,802,887	140,948	92,537	154,890	780,308	146,864	2,029,634	341,671	113,487	0	15,567,627
CY3	1,076,993	70,950	103,009	342,534	1,043,577	181,775	2,078,913	439,574	171,783	0	23,636,607
YK1	3,975,469	143,865	278,957	418,914	1,327,922	433,379	4,356,065	740,771	273,545	0	37,310,411
YK2	2,496,856	104,354	132,545	330,588	584,479	148,006	2,625,391	222,451	98,620	0	21,605,297
YK3	2,040,259	92,021	72,883	80,794	538,848	122,537	3,129,987	197,924	101,599	0	28,880,074

Table S1. Statistics for classified small RNA from cattleyak and yak testis miRNA-seq data.

MiRNA name	Sample name	Location	Sequence	Length	Genome hits	Hairpin length	Hairpin % G/C content	Minimum free energy	Adjusted MFE	Randfold p-value
Bta-novel-miR-1	YK1, YK3	chr16:18872291-18872312:[-]	AAAAAACCGAGTGGACTTTTTG	22	1	110	44.54545	-51.2	-46.5455	0.009901
Bta-novel-miR-2	CY2	chr23:24510595-24510617:[-]	AAAATCTGTATGGCATGATAACT	23	2	161	32.29814	-40.79	-25.3354	0.019802
Bta-novel-miR-3	CY1	chr13:38147531-38147550:[-]	AAATGCCCTGCAGTCTGCTG	20	1	66	54.54545	-21.9	-33.1818	0.049505
Bta-novel-miR- 4	CY1,2, YK1,2	chr12:50716633-50716654:[+]	AACGTAGAATATGCAGCAGGAC	22	2	52	40.38462	-13.2	-25.3846	0.039604
Bta-novel-miR- 5	CY1,2	chr19:23217212-23217234:[-]	AACTCTGTGTGGTGCTGAGGCTC	23	1	94	52.12766	-33.9	-36.0638	0.049505
Bta-novel-miR- 6	CY2 YK1,2	NW_001503906.2:21904-21925:[+]	AACTGTTAGGAGGCTTGGCTGC	22	1	124	53.22581	-52.79	-42.5726	0.009901
Bta-novel-miR- 7	CY3	chr8:11265865-11265886:[-]	AAGGACCAGTGCGAGAAGTGCC	22	1	151	62.25166	-67.6	-44.7682	0.09901
Bta-novel-miR- 8	CY1	chr11:317742-317763:[-]	AATACTGAGTTGGCTAGAAAGT	22	1	79	43.03797	-36	-45.5696	0.009901
Bta-novel-miR- 9	CY1,2	chr4:101889395-101889417:[+]	ACACCACCCTGAACGCGCCCGAT	23	1	119	56.30252	-48.8	-41.0084	0.019802
Bta-novel-miR- 10	CY1	chr6:72372959-72372976:[-]	ACAGGATTGACAGATTGA	18	4	87	45.97701	-28	-32.1839	0.049505
Bta-novel-miR- 11	CY1,2 YK1,2,3	chrX:17249212-17249233:[]	ACATAGTGCCTTTCTGGGTTGT	22	1	79	45.56962	-31.9	-40.3797	0.009901
Bta-novel-miR- 12	CY2, YK1,2	chr14:13270477-13270499:[-]	ACATGCAAAGGATCTCCCTGACT	23	3	62	50	-24.6	-39.6774	0.019802
Bta-novel-miR- 13	CY1,2,3 YK1,2,3	chr8:102341314-102341336:[+]	ACCTCCCGTGGAGCAGAAGGGCA	23	10	158	51.26582	-56.3	-35.6329	0.069307
Bta-novel-miR- 14	YK3	chr12:14453535-14453556:[-]	ACGCATCTATTTGACAGACCTG	22	2	136	42.64706	-46.2	-33.9706	0.009901
Bta-novel-miR- 15	YK2	NW_001493008.1:277555-277576:[-]	ACGGGCGAGGGCATGGACGAGA	22	6	88	62.5	-36.7	-41.7045	0.049505
Bta-novel-miR- 16	CY2	chr21:65937137-65937159:[+]	ACGTGTCAGGAGCTCTGAGGTCC	23	1	73	47.94521	-22.8	-31.2329	0.069307
Bta-novel-miR- 17	YK3	NW_001508850.2:265452-265474:[+]	ACTAAGTCAAGTAGAACCAAGAA	23	2	84	47.61905	-42.9	-51.0714	0.009901
Bta-novel-miR- 18	CY2, YK1,2	chr14:13104096-13104117:[-]	ACTTCAGAGACTGGACTGTGCC	22	8	100	60	-41.1	-41.1	0.089109
Bta-novel-miR- 19	CY2	NT_186281.1:1869-1891:[+]	ACTTTTGCCCCTAGTAACGGACT	23	1	54	53.7037	-23.22	-43	0.009901
Bta-novel-miR- 20	CY2	chr29:51173239-51173260:[+]	AGACTCAGAGCCTCCAGAGCCC	22	1	114	58.77193	-49.13	-43.0965	0.009901
Bta-novel-miR- 21	CY1,2,3 YK1,2,3	chr29:46149483-46149504:[-]	AGGGACAAGTGGCGTTCAGCCA	22	4	92	63.04348	-39.3	-42.7174	0.039604
Bta-novel-miR- 22	CY1,2 YK3	chr8:37767631-37767649:[-]	AGGGGCTCTCGCTTCTGGC	19	5	96	78.125	-54.7	-56.9792	0.079208
Bta-novel-miR- 23	CY2	chr10:77712450-77712468:[-]	AGGTGTCAGAAAAGTTACC	19	6	132	40.90909	-37.72	-28.5758	0.079208
Bta-novel-miR- 24	CY2	chr19:54656312-54656333:[+]	AGTTTCTGGACATCGAGGACCC	22	1	93	56.98925	-40.9	-43.9785	0.019802
Bta-novel-miR- 25	YK1,2	chr5:17677483-17677502:[+]	ATACATAGGGCTTGTTTTCC	20	3	153	39.21569	-48	-31.3725	0.009901
Bta-novel-miR- 26	YK2,3	chr8:103403919-103403941:[+]	ATCATTCTTTCACTCTGCTTCCT	23	1	99	49.49495	-32.2	-32.5253	0.039604
Bta-novel-miR- 27	CY1,2,3	chr21:60626164-60626186:[-]	ATCTGTAGTCTCGGCGTCGCACT	23	1	59	57.62712	-22.1	-37.4576	0.079208

Bta-novel-miR- 28	CY2, YK1	NW_001506628.1:8333-8355:[+]	ATGTGGGCTAGTTTCGGACAAGG	23	4	84	47.61905	-29	-34.5238	0.009901
Bta-novel-miR- 29	CY1,2,3 YK1,2,3	NT_184731.1:59559-59579:[-]	ATTGGCATTTCTTAGAGTGGA	21	1	126	43.65079	-55.6	-44.127	0.009901
Bta-novel-miR- 30	CY1, YK3	NT_185358.1:947-967:[+]	ATTGGCGCTCTTTTGAGTGTG	21	1	274	44.89051	-94.3	-34.4161	0.009901
Bta-novel-miR- 31	YK1	chr3:73232436-73232458L:[-]	ATTTTAAAATGGCACTGAGACCT	23	1	134	35.07463	-36.3	-27.0896	0.029703
Bta-novel-miR- 32	YK1	chr1:82008274-82008296:[-]	CACCAAAAGTTTGTTATTGGGGG	23	1	91	39.56044	-31.3	-34.3956	0.009901
Bta-novel-miR- 33	CY1,2,3 YK1,2,3	chr10:16545156-16545176:[+]	CACCTAGCACTCGCTCGCACC	21	2	139	67.6259	-65.6	-47.1942	0.019802
Bta-novel-miR- 34	CY1	chr14:13107101-13107119:[-]	CACCTGTGACTCCTGCAAC	19	2	73	56.16438	-25.82	-35.3699	0.039604
Bta-novel-miR- 35	YK1	chr12:67657872-67657893:[-]	CATACATAGGGCTTGTTTTCCA	22	3	175	41.14286	-49.1	-28.0571	0.079208
Bta-novel-miR- 36	CY1,2,3 YK1,2,3	chr7:39694724-39694746:[-]	CATGTCCTGCAATTCTGAAGGGA	23	1	212	51.88679	-80.5	-37.9717	0.049505
Bta-novel-miR- 37	CY2	chr3:23597313-23597332:[+]	CCATTGATCGCCAGGGTTGA	20	12	78	58.97436	-32.8	-42.0513	0.029703
Bta-novel-miR- 38	CY2 YK1	chr2:130337511-130337532:[-]	CCCAACGTGGATACCCCGGGAG	22	1	55	67.27273	-26.2	-47.6364	0.079208
Bta-novel-miR- 39	CY1, YK1,2,3	chr3:9025265-9025283:[-]	CCCGCCCCCCCGCCTCCCC	19	6	97	75.25773	-58.9	-60.7216	0.019802
Bta-novel-miR- 40	CY1, YK1,2,3	chr3:120901931-120901948:[]	CCCGCGAGGGGGCGGGGC	18	5	99	73.73737	-54.6	-55.1515	0.089109
Bta-novel-miR- 41	YK3	NT_186807.1：12259-12280:[+]	CCCGCGGCCCTGTCCCGCGGCC	22	2	253	78.65613	-161.81	-63.9565	0.029703
Bta-novel-miR- 42	CY1,2 YK1	chr8:96283004-96283021:[-]	CCGGCGGCGGCGGCGACT	18	3	91	65.93407	-45.1	-49.5604	0.009901
Bta-novel-miR- 43	CY1, YK2	NT_186807.1:11785-11806:[+]	CGCGTGTGCGCGCGGTCGGCCC	22	3	114	81.57895	-70.5	-61.8421	0.09901
Bta-novel-miR- 44	CY2, YK3	chr3:8805035-8805056:[-]	CGGCGCTCTCACCGCCGCGGCC	22	7	111	72.97297	-61	-54.955	0.039604
Bta-novel-miR- 45	CY2	NW_001503883.1:46673-46694:[-]	CGGGGACGCAACTCATCTCGCC	22	2	108	61.11111	-42.1	-38.9815	0.029703
Bta-novel-miR- 46	YK1,2	chr8:62283764-62283786:[-]	CGGGGCTCATTCTCAGCACGTCT	23	1	96	60.41667	-37.8	-39.375	0.009901
Bta-novel-miR- 47	CY1,2 YK2,3	chr10:103609388-103609405:[+]	CGGGGCTGGGCGCGCGCC	18	2	86	79.06977	-54.5	-63.3721	0.019802
Bta-novel-miR- 48	CY1	NT_186807.1:12611-12632:[+]	CGTCCCGGGCGTGCGGTGGGAC	22	2	143	79.02098	-87	-60.8392	0.089109
Bta-novel-miR- 49	YK1	chr10:89648986-89649007:[+]	CTCATAATTTTTGGAGGCTTTG	22	2	59	44.0678	-19.2	-32.5424	0.039604
Bta-novel-miR- 50	CY1 YK2,3	chr14:13179610-13179628:[-]	CTCCACCCTTGTTGGGCAG	19	3	141	50.35461	-64.39	-45.6667	0.009901
Bta-novel-miR- 51	YK2	chr8:7406580-7406598:[-]	CTCCCGGGTTTCGGCACCA	19	6	136	66.91176	-69	-50.7353	0.029703
Bta-novel-miR- 52	CY1	chr12:21236132-21236154:[+]	CTCCTGCATCGAAAGTGATTGTG	23	3	55	54.54545455	-18.9	-34.3636 3636	0.009901
Bta-novel-miR- 53	CY1	chr13:67894471-67894493[-]	CTCTGGACTCAAGTTCTGAGCC	23	3	72	56.94444444	-29.4	-40.8333 3333	0.039604
Bta-novel-miR- 54	CY2	chr7:51656870-51656891[-]	CTGAAGACTGGAGTGCTCAGCC	22	1	104	57.69230769	-45.6	-43.8461 5385	0.049505

Bta-novel-miR- 55	YK2	chr19:40379828-40379850[-]	CTGCAGATCAAGAGGTCCCCGGT	23	10	84	52.38095238	-27	-32.14285714	0.029703
Bta-novel-miR- 56	YK1	chr15:54113191-54113212[]	CTTCAGTGATGACACGATGACG	22	1	61	50.81967213	-22.7	-37.21311475	0.009901
Bta-novel-miR- 57	YK1	chr5:25967046-25967068[+]	CTTGCAAACTGTCTGAATTTGCC	22	1	61	50.81967213	-22.7	-37.21311475	0.009901
Bta-novel-miR- 58	CY3, YK3	chr17:5453362-5453379[+]	CTTTTCTTTGTGAAGGGC	18	7	79	36.70886076	-20.5	-25.94936709	0.089109
Bta-novel-miR- 59	CY1	chr19:56436571-56436592[-]	AAAACAATCTGTTCTGAGGCT	22	1	105	52.38095238	-36.8	-35.04761905	0.019802
Bta-novel-miR- 60	YK1	chr20:67344124-67344146[-]	GAAAATGATGAATTCTGGGGCGC	23	1	163	38.65030675	-44.1	-27.05521472	0.059406
Bta-novel-miR- 61	CY1,2 YK2,3	chr19:8789290-8789309[-]	GAGAACGTAATCTGAGTGGT	20	2	124	42.74193548	-41.9	-33.79032258	0.009901
Bta-novel-miR- 62	CY1,2	chr19:17961259-17961279[+]	GAGCACTGTTCGTGACCTGCT	21	1	149	56.37583893	-60.6	-40.67114094	0.079208
Bta-novel-miR- 63	CY2	chr2:124743810-124743827[-]	GATAGTCAAGTTCTGATC	18	1	126	38.0952381	-39.5	-31.34920635	0.009901
Bta-novel-miR- 64	CY3	chr29:42383956-42383976[-]	GATATTGACATCTCTGGACCC	21	7	160	52.5	-60.8	-38	0.019802
Bta-novel-miR- 65	CY1	NW_001497819.1_608-627[+]	GATATTGACATCTCTGGACC	20	7	186	53.22580645	-69.5	-37.3655914	0.019802
Bta-novel-miR- 66	YK2,3	chr13:52907883-52907904[-]	GATGTCTCCATGTCTCTGAGCA	22	1	92	46.73913043	-25.5	-27.7173913	0.089109
Bta-novel-miR- 67	YK2	chr3:24239329-24239348[-]	GCACTCTGGACTTTGAATCC	20	14	71	53.52112676	-24.4	-34.36619718	0.079208
Bta-novel-miR- 68	CY2	chr7:17175965-17175986[-]	GCAGCCAAGAAGGACTGAGCCC	22	4	87	57.47126437	-35.7	-41.03448276	0.009901
Bta-novel-miR- 69	CY2,3 YK1,2	chr2:72367568-72367585[+]	GCATGGGTGGTTCAGTGG	18	14	112	48.21428571	-35.6	-31.78571429	0.039604
Bta-novel-miR- 70	CY1	NW_001506399.2_8831-8853[-]	GCCCTGACCTTCGGTCTCCTGTC	23	4	94	71.27659574	-50.5	-53.72340426	0.059406
Bta-novel-miR- 71	CY1 YK1,3	chr2:101406972-101406993[+]	GCGTTGGTGGTATAGTGGTGAG	22	11	51	49.01960784	-17.3	-33.92156863	0.019802
Bta-novel-miR- 72	CY2	chr6:93932574-93932596[+]	GCTGAGAATTGTGGAGCCATACA	23	11	53	43.39622642	-18.3	-34.52830189	0.009901
Bta-novel-miR- 73	YK1	chr19:26645281-26645302[+]	GCTGCAGGATGGAGAATTTACC	22	2	137	50.3649635	-39.62	-28.91970803	0.059406
Bta-novel-miR- 74	CY2	chr4:89153590-89153608[-]	GGAAACTCTGGTGGAGGTC	19	4	152	45.39473684	-50.3	-33.09210526	0.019802
Bta-novel-miR- 75	YK3	chr21:52035490-52035510[-]	GGCCGTGTGCCGCTCGTTGCC	21	3	61	83.60655738	-49.1	-80.49180328	0.009901
Bta-novel-miR- 76	CY1	chr6:78036670-78036687[+]	GGCTGGTCCGATGGTAGT	18	2	94	43.61702128	-32.61	-34.69148936	0.009901

Bta-novel-miR- 77	CY3	chr3:17413311-17413332[-]	GGGCATACTTGTAGACCTTGCC	22	1	59	57.62711864	-30.3	-51.3559322	0.019802
Bta-novel-miR- 78	CY2 YK1,2	NT_183129.1_192-213[-]	GGGCCGTGTGCCGCTCGTTGCC	22	3	55	81.81818182	-40	-72.72727273	0.009901
Bta-novel-miR- 79	YK1	chr4:91654916-91654933[-]	GGGCGCGCGCCGCGGCTG	18	2	141	77.30496454	-81	-57.44680851	0.079208
Bta-novel-miR- 80	CY1	chr23:30072999-30073018[+]	GGGGGTGTAGCTCAGTGGTA	20	16	138	52.89855072	-54.1	-39.20289855	0.009901
Bta-novel-miR- 81	YK2	chr23:29130547-29130567[+]	GGGGGTGTAGCTCAGTGGTAG	21	15	132	56.06060606	-50.8	-38.48484848	0.089109
Bta-novel-miR- 82	YK1	chr19:28098913-28098934[-]	GGGTAGTTGAGTGTAGTATATT	22	1	141	50.35460993	-49.2	-34.89361702	0.089109
Bta-novel-miR- 83	CY1	chr12:41034461-41034478[-]	GGTGGCGGGGGGAGGTCT	18	3	90	50	-34.12	-37.91111111	0.009901
Bta-novel-miR- 84	CY1,3	chr24:23779081-23779102[+]	GTCACCCCATTGATCGCCAGGG	22	13	53	58.49056604	-18.6	-35.09433962	0.039604
Bta-novel-miR- 85	CY1	chr18:24839516-24839535[+]	GTCAGGATGGCCGAGCGGTC	20	6	132	57.57575758	-49.42	-37.43939394	0.019802
Bta-novel-miR- 86	CY1	chr15:42769991-42770012[-]	GTCGAGGCTAGAGTCACGCTTG	22	1	84	51.19047619	-33.7	-40.11904762	0.009901
Bta-novel-miR- 87	YK2	NW_001502870.1_32207-32229[-]	GTGTAGACTTTTGGATCGCAGCC	23	11	152	46.71052632	-44.14	-29.03947368	0.019802
Bta-novel-miR- 88	CY1	chr7:33295607-33295624[-]	GTTTAGGTGGCAGAGCCC	18	1	86	50	-30.7	-35.69767442	0.019802
Bta-novel-miR- 89	YK1	chr6:101595666-101595686[+]	TAAGGGACCTTGGATACAACT	21	1	96	52.08333333	-31.31	-32.61458333	0.049505
Bta-novel-miR- 90	YK2	chr23:8347899-8347921[-]	TAATCCAGATATCTTGATCCACC	23	1	50	50	-16.5	-33	0.039604
Bta-novel-miR- 91	YK3	chr28:32838063-32838084[+]	TACATTTCTGGCCTAATGCATC	22	1	67	37.31343284	-16.8	-25.07462687	0.09901
Bta-novel-miR- 92	YK2,3	chr7:13844661-13844683[+]	TACCAGTTCTACCTGGCTAGGAT	23	1	50	60	-18.8	-37.6	0.039604
Bta-novel-miR- 93	YK3	chr10:47899820-47899840[-]	TACTGGGCAGAAACTGATTTC	21	1	88	50	-28.4	-32.27272727	0.019802
Bta-novel-miR- 94	YK1	ChrX:17248143-17248163[+]	TACTGTGCCCTGGATGGGTAG	21	1	82	54.87804878	-36.6	-44.63414634	0.009901
Bta-novel-miR- 95	CY1	ChrX:17253954-17253974[+]	TACTGTGCCCTGTATGGGTAG	21	1	111	53.15315315	-49	-44.14414414	0.009901
Bta-novel-miR- 96	CY1	ChrX:17221885-17221902[+]	TACTGTGCCTTGAATGGG	18	4	82	51.2195122	-37.5	-45.73170732	0.009901
Bta-novel-miR- 97	CY1	ChrX:17224842-17224860[+]	TACTGTGCCTTGAATGGGT	19	4	73	52.05479452	-31.6	-43.28767123	0.009901
Bta-novel-miR- 98	YK2	chr21:29889678-29889700[+]	TACTTGGAAGAACTCATTTTAAA	23	2	109	48.62385321	-35.2	-32.29357798	0.079208
Bta-novel-miR- 99	YK3	chr10:102366159-102366181[-]	TAGCCTGTGAACTTTGTGGTTTC	23	2	143	38.46153846	-39.8	-27.8321	0.039604

									6783	
Bta-novel-miR- 100	YK3	chr8:18009183-18009205[+]	TAGGATGCAAAATCACTGCTGCT	23	1	50	48	-15.3	-30.6	0.039604
Bta-novel-miR- 101	YK2	chr24:43778631-43778653	TAGTTAGGGCAGTGTGAGTCAGG	23	1	53	47.16981132	-13.5	-25.4716<br>9811	0.089109
Bta-novel-miR- 102	YK3	chr8:18011864-18011886[+]	TATAGATAGAGCGTCCAGCAGCT	23	1	154	55.19480519	-63.2	-41.0389<br>6104	0.089109
Bta-novel-miR- 103	YK3	chr8:18023359-18023379[+]	TATTCAGACTTGGGTCAAGCC	21	1	119	57.98319328	-49.3	-41.4285<br>7143	0.059406
Bta-novel-miR- 104	YK2,3	chr21:9058366-9058388[+]	TCAAAAGGTTCTGTCTTCTGGCT	23	1	100	49	-35.4	-35.4	0.019802
Bta-novel-miR- 105	YK3	chr24:43826159-43826179[-]	TCAAGATTGAGTTTCTGAGCC	21	1	73	52.05479452	-29.4	-40.2739<br>726	0.019802
Bta-novel-miR- 106	YK3	chr3:15252738-15252755[+]	TCAAGTAGAACCAAGAAA	18	5	70	48.57142857	-37.6	-53.7142<br>8571	0.009901
Bta-novel-miR- 107	CY1,2<br>YK1,2	NT_186680.1_401-422[+]	TCAAGTCCCTGTTCGGGCGCCA	22	16	104	51.92307692	-38	-36.5384<br>6154	0.039604
Bta-novel-miR- 108	CY1,2<br>YK1	chr7:11067820-11067842[+]	TCAAGTGATGGAGATGACAACCT	23	1	101	59.40594059	-34.3	-33.9603<br>9604	0.009901
Bta-novel-miR- 109	YK2	chr7:99031284-99031305[+]	TCAGAACTCTGCAGCATGACCC	22	9	142	47.88732394	-49.1	-34.5774<br>6479	0.079208
Bta-novel-miR- 110	YK3	chr12:29284074-29284094[+]	TCATTTGTGGAACCCTGGCGC	21	1	81	49.38271605	-26.3	-32.4691<br>358	0.09901
Bta-novel-miR-111	YK3	chr10:59259806-59259824[-]	TCGCATTTGAGCTGGACCT	19	1	60	55	-22.5	-37.5	0.019802
Bta-novel-miR- 112	CY1,2,3<br>YK2	chr29:43141286-43141306[+]	TCGCTTTTTGATCCTTCGATG	21	5	102	40.19607843	-33.5	-32.8431<br>3725	0.009901
Bta-novel-miR- 113	CY2	NW_001503085.2_25882-25903[-]	TCTGCAGAAGCCTCTTACATAA	22	1	57	47.36842105	-21.1	-37.0175<br>4386	0.049505
Bta-novel-miR- 114	YK3	NW_001505770.1_2174-2196[+]	TCTGTAGTCCTGAATAGATATCA	23	2	62	40.32258065	-17.1	-27.5806<br>4516	0.059406
Bta-novel-miR- 115	YK3	NT_185358.1_6106-6124[+]	TCTGTCACCTTCTTGAGTG	19	1	166	46.98795181	-76.3	-45.9638<br>5542	0.009901
Bta-novel-miR- 116	YK3	chr19:56252028-56252050[-]	TCTGTCTGTAGTGCAGGAGACCT	23	9	113	38.9380531	-35.87	-31.7433<br>6283	0.019802
Bta-novel-miR- 117	CY2	chr5:10822635-10822656[+]	TCTTAAAGGCACGAGAGGACAG	22	1	71	53.52112676	-31.5	-44.3661<br>9718	0.019802
Bta-novel-miR- 118	CY3	chr10:58883419-58883440[+]	TCTTAAGATTTGGTGCAATATG	22	3	87	42.52873563	-27.8	-31.9540<br>2299	0.009901
Bta-novel-miR- 119	YK2,3	chr18:38632177-38632199[+]	TCTTAGAATTCTCACTTGGAAGA	23	1	110	61.81818182	-45.29	-41.1727<br>2727	0.019802
Bta-novel-miR- 120	YK1	chr23:30042338-30042360[-]	TCTTTACAGGCCTCGTGGAACCC	23	2	98	43.87755102	-27.8	-28.3673<br>4694	0.009901
Bta-novel-miR- 121	YK3	chr9:34664277-34664297[+]	TCTTTGAGACTCCACGGACTG	21	14	103	51.45631068	-38.4	-37.2815<br>534	0.029703
Bta-novel-miR- 122	CY2	chr29:662873-662895[+]	TGAACTAAAACTTAATTCTGATA	23	3	119	34.45378151	-31	-26.0504	0.019802

	YK1,2								2017	
Bta-novel-miR- 123	YK3	chr17:19180002-19180024[+]	TGAAGAAACTGAGGCACAAAGAG	23	9	61	34.42622951	-19	-31.14754098	0.009901
Bta-novel-miR- 124	CY1<br>YK1,2	NW_001508724.2_22332-22352[-]	TGAGCACACCTGCCTGAGCAG	21	1	278	48.56115108	-98.8	-35.53956835	0.009901
Bta-novel-miR- 125	YK3	chr8:12932677-12932696[+]	TGAGGATATCAGAGAAACTG	20	1	75	45.33333333	-21.9	-29.2	0.09901
Bta-novel-miR- 126	CY1<br>YK1	chr29:662550-662571[+]	TGAGGTCTATCCCGATGGGGCT	22	1	56	46.42857143	-18.2	-32.5	0.029703
Bta-novel-miR- 127	CY2<br>YK1	chr1:84407381-84407403[-]	TGATGGAACTGAGGATCTGAGGA	23	1	126	56.34920635	-49.3	-39.12698413	0.09901
Bta-novel-miR- 128	CY2,3<br>YK2	NT_184731.1_63275-63297[-]	TGATTGGTACTTCTTAGAGTGGA	23	1	130	47.69230769	-63.17	-48.59230769	0.009901
Bta-novel-miR- 129	CY1<br>YK2,3	NW_001505770.1_358-377[+]	TGATTGGTCTTTTGAATGAC	20	9	166	41.56626506	-54	-32.53012048	0.009901
Bta-novel-miR- 130	YK3	NW_001502712.2_78872-78894[+]	TGCAACTGAAGATCCTGCATGCC	23	12	71	49.29577465	-26.1	-36.76056338	0.009901
Bta-novel-miR- 131	YK3	chr24:43821835-43821856[-]	TGCACATCCAGAGGGCTGTGTA	22	1	155	65.16129032	-88.1	-56.83870968	0.009901
Bta-novel-miR- 132	YK3	chr5:108928753-108928775[+]	TGCAGGTGGATTCTGTACCAACT	23	15	51	52.94117647	-15.8	-30.98039216	0.09901
Bta-novel-miR- 133	CY2<br>YK1,2	chr4:18492683-18492704[-]	TGCCAGGTGGGGAGTTTGACTG	22	8	67	58.20895522	-27	-40.29850746	0.009901
Bta-novel-miR- 134	YK2	chr23:8333587-8333609[-]	TGCCCTGGGTTGATGTAAGAGCT	23	1	153	48.36601307	-51.16	-33.4379085	0.019802
Bta-novel-miR- 135	YK3	chr10:59243811-59243833[-]	TGCCTGTGGACAGATGTATACCC	23	1	180	47.22222222	-60.8	-33.77777778	0.009901
Bta-novel-miR- 136	YK2	chr12:29260374-29260396[+]	TGCCTTGACGTTCTCTGTGTTCC	23	1	107	54.20560748	-44.6	-41.68224299	0.029703
Bta-novel-miR- 137	YK3	chr23:8322655-8322676[-]	TGCCTTTTGTCAGAGCCTGAGC	22	1	65	53.84615385	-27.8	-42.76923077	0.039604
Bta-novel-miR- 138	CY1	chr23:30930402-30930424[+]	TGCGAAGAATAGGAAGCTGGCCT	23	1	71	46.47887324	-21.44	-30.1971831	0.069307
Bta-novel-miR- 139	YK3	chr21:13755899-13755917[-]	TGCTAAGTGACTAGACACC	19	1	153	41.83006536	-42	-27.45098039	0.039604
Bta-novel-miR- 140	YK3	chr15:75962626-75962648[-]	TGCTAGAGAAATCGGAGAGAATC	23	1	115	40	-32.81	-28.53043478	0.029703
Bta-novel-miR- 141	CY1	ChrX:84354759-84354780[+]	TGCTCCTGCTTGAACATAGTCT	22	1	75	46.66666667	-35.9	-47.86666667	0.009901
Bta-novel-miR- 142	CY1,2	chr20:61846085-61846102[-]	TGGGAATGATGATTTCTC	18	2	78	33.33333333	-22.3	-28.58974359	0.029703
Bta-novel-miR- 143	YK3	chr10:59243054-59243075[-]	TGGGAGTTGCAGGCCACTATAC	22	1	138	45.65217391	-48.6	-35.2173913	0.029703
Bta-novel-miR- 144	YK3	chr17:68154352-68154371[-]	TGGGCCTGAAGTCTGTTTTG	20	1	141	43.97163121	-44.4	-31.4893	0.029703

									617	
Bta-novel-miR- 145	YK2	NW_001503331.1_59376-59397[+]	TGGTGACTGTCCTCGCCGTGCC	22	6	74	67.56756757	-41.3	-55.8108 1081	0.009901
Bta-novel-miR- 146	CY2,3 YK1	NW_001502565.1_23006-23024[-]	TGTCCCTGTTCGGGCGCCA	19	9	81	67.90123457	-38.2	-47.1604 9383	0.049505
Bta-novel-miR- 147	YK3	chr28:32833691-32833713[+]	TGTCTGCAGACTGGCCACCCTGA	23	1	159	57.86163522	-68.4	-43.0188 6792	0.039604
Bta-novel-miR- 148	YK2,3	NW_001502911.2_20709-20729[+]	TGTGATGAGATATCAAGAACC	21	8	146	50	-57.64	-39.4794 5205	0.009901
Bta-novel-miR- 149	YK3	chr19:56279222-56279243[+]	TGTGGACTTTTCCAGTTCGGCC	22	1	183	58.46994536	-82.1	-44.8633 8798	0.019802
Bta-novel-miR- 150	YK3	chr16:29541773-29541794[-]	TGTGGCCTCTGGGTGTGTACCC	22	1	140	49.28571429	-55.3	-39.5	0.019802
Bta-novel-miR- 151	YK3	chr28:32822141-32822160[+]	TGTTTACTGATTAGGAAATA	20	1	99	36.36363636	-28.6	-28.8888 8889	0.009901
Bta-novel-miR- 152	YK2	NW_001503327.2_21068-21089[+]	TTAGTATCTTGGATGGGAGACC	22	14	59	50.84745763	-23	-38.9830 5085	0.029703
Bta-novel-miR- 153	YK3	chr16:3016477-3016498[+]	TTATGTCCGACTCTTGCAACCC	22	6	85	37.64705882	-21.4	-25.1764 7059	0.069307
Bta-novel-miR- 154	CY1,2 YK1,2,3	NT_184731.1_45171-45191[-]	TTCATAGGAAGGTGTCATTCA	21	1	126	43.65079365	-55.6	-44.1269 8413	0.009901
Bta-novel-miR- 155	YK3	chr5:81025290-81025311[-]	TTCCAGGACTTTCTTTCAACCT	22	1	193	47.15025907	-67.1	-34.7668 3938	0.079208
Bta-novel-miR- 156	YK3	chr1:78325647-78325669[+]	TTCCAGTTGAGCTGTTTCAAACC	23	13	97	45.36082474	-32	-32.9896 9072	0.009901
Bta-novel-miR- 157	YK1,2	chr3:32871458-32871475[[-]	TTCCCGGCCCATGCACCA	18	13	81	46.91358025	-30.6	-37.7777 7778	0.009901
Bta-novel-miR- 158	CY2 YK2	chr13:54407150-54407171[-]	TTCTCAGGTTGGACAGTCCTGA	22	1	146	63.01369863	-65.71	-45.0068 4932	0.059406
Bta-novel-miR- 159	YK3	chr8:18014879-18014901[+]	TTCTCATGACTGTGGGATTCCCC	23	1	81	46.91358025	-26.5	-32.7160 4938	0.029703
Bta-novel-miR- 160	CY2,3	chr3:107182820-107182841[+]	TTCTCTTCAGATCGTATAAATC	22	5	60	38.33333333	-16	-26.6666 6667	0.039604
Bta-novel-miR- 161	YK3	chr23:8331616-8331638[-]	TTCTGGTCTCTGGAAGCTTGGCA	23	1	130	60.76923077	-62.8	-48.3076 9231	0.019802
Bta-novel-miR- 162	YK3	chr12:29274750-29274772[+]	TTCTTCCTGTTGTTTGCGATCAC	23	1	68	48.52941176	-25.2	-37.0588 2353	0.019802
Bta-novel-miR- 163	CY1	NW_001502372.2_16452-16470[-]	TTGGCATGTCTTGGAATGA	19	1	122	47.54098361	-50.2	-41.1475 4098	0.009901
Bta-novel-miR- 164	YK2,3	chr23:8350407-8350428[+]	TTGTAGATAACTTGGATGTGCC	22	1	107	47.6635514	-52.2	-48.7850 4673	0.009901
Bta-novel-miR- 165	YK3	ChrX:57562783-57562805[-]	TTGTGTCTGACTCTGCGACCCCC	23	11	79	48.10126582	-23.8	-30.1265 8228	0.019802

Bta-novel-miR- 166	YK1	chr9:100277893-100277915[-]	TTTCCCATTTGACTACCACATTA	23	1	88	42.04545455	-23	-26.1363 6364	0.049505
Bta-novel-miR- 167	CY2 YK2	chr11:40070993-40071011[+]	TTTCCCGGCCCATGCACCA	19	12	88	50	-33.5	-38.0681 8182	0.009901
Bta-novel-miR- 168	YK1	chr17:68324312-68324333[+]	TTTGCCCGGATTTTTCTGTAAC	22	1	195	36.41025641	-62.37	-31.9846 1538	0.009901

Table S2. Statistics for novel miRNAs identified from testis of cattleyak and yak.

MiRNA	Total score	Total energy	Gene symbol	Gene description
bta-miR-126-3p	174	-24.64	ZBTB20	zinc finger and BTB domain containing 20
bta-miR-130b	179	-31.3	TMEM194A	Bos taurus transmembrane protein 194A
	179	-26.11	ERAP1	endoplasmic reticulum aminopeptidase 1
	321	-47.22	DLG5	Bos taurus discs, large homolog 5 (Drosophila)
bta-miR-135a	175	-20.59	THRSP	Bos taurus thyroid hormone responsive
	176	-22.47	ANKRD17	ankyrin repeat domain 17
	177	-21.47	TMC2	Bos taurus transmembrane channel-like 2
	178	-23.59	PALM3	paralemmin 3
	180	-25.04	CUBN	Bos taurus cubilin (intrinsic factor-cobalamin receptor)
	182	-22.55	CSPP1	centrosome and spindle pole associated protein 1
	185	-21.19	RABGAP1L	Bos taurus RAB GTPase activating protein 1-like
	304	-42.05	ZNF2	Bos taurus zinc finger protein 2
	312	-47.58	ZNF596	Bos taurus zinc finger protein 596
	314	-43.26	ZNF154	zinc finger protein 154
	322	-46.3	ZNF76	zinc finger protein 76
	323	-49.58	ZNF169	zinc finger protein 169
	326	-44.44	ZNF226	Bos taurus zinc finger protein 226
	332	-45.71	ZNF167	Bos taurus zinc finger protein 167
	335	-43.08	MTF1	Bos taurus metal-regulatory transcription factor 1
	337	-44.8	ZBTB24	zinc finger and BTB domain containing 24
	337	-45.9	MYNN	Bos taurus myoneurin
	800	-110	ZNF845	zinc finger protein 845
bta-miR-19b	177	-21.12	SPEF2	sperm flagellar 2
	181	-22.07	CCNT2	cyclin T2
bta-miR-2318	186	-21.6	RABEP1	rabaptin, RAB GTPase binding effector protein 1
	315	-45.18	ZNF133	Bos taurus zinc finger protein 133
bta-miR-2332	175	-23.64	STOM	stomatin
	175	-20.26	HAUS5	HAUS augmin-like complex, subunit 5
	177	-25.03	IFI35-201	Bos taurus interferon-induced protein 35 (IFI35)
	179	-24.44	IL6ST	interleukin 6 signal transducer
	179	-26.79	CDK2	Bos taurus cyclin-dependent kinase 2
	186	-25.4	RP2	Bos taurus retinitis pigmentosa 2 (X-linked recessive)
bta-miR-2419-5p	174	-24.94	PRKCD	protein kinase C, delta
	317	-50.07	FLYWCH1	FLYWCH-type zinc finger 1
bta-miR-2435	174	-31.81	AGAP3	ArfGAP with GTPase domain, ankyrin repeat and PH domain 3
bta-miR-2483-5p	176	-22.68	ME2	Bos taurus malic enzyme 2, NAD(+)-dependent, mitochondrial
	179	-28.69	ENO4	enolase family member 4
	320	-48.37	LRP12	low density lipoprotein receptor-related protein 12
	324	-45.71	GOLGA4	golgin A4
bta-miR-2484	178	-21.32	RHCG	Bos taurus Rh family, C glycoprotein
	178	-22.48	DDX50	Bos taurus DEAD (Asp-Glu-Ala-Asp) box polypeptide 50

	180	-25.73	ASB15	Bos taurus ankyrin repeat and SOCS box containing 15
	180	-23.67	CHST2	carbohydrate (N-acetylglucosamine-6-O) sulfotransferase 2
bta-miR-503-3p	174	-21.99	HMGN4	Bos taurus high mobility group nucleosomal binding domain 4
	347	-51.32	AFF2	AF4/FMR2 family, member 2
bta-miR-592	174	-27.36	ATP5A1	ATP synthase, H+ transporting, mitochondrial F1 complex, alpha subunit 1
	175	-20.09	TPP2	tripeptidyl peptidase II
bta-miR-9-5p	178	-26.64	NIN	ninein (GSK3B interacting protein)
	323	-45.21	DOCK7	dedicator of cytokinesis 7
	340	-47.12	KANK1	KN motif and ankyrin repeat domains 1

Table S3.Target genes for the first 13 differentially expressed known miRNAs in testis from cattleyak (CY) versus yak (YK) are shown, using target mining software of miRanda. MiRNAs with ＞300 predicted targets in genome were excluded, whereas target genes with ≧174 total score were shown.

MiRNA	Total score	Total energy	Gene symbol	Gene description
bta-novel-miR-10	173	-24.3	TRIM33	tripartite motif containing 33
	172	-25.57	SIK2	salt-inducible kinase 2
	178	-23.19	MAP3K8	Bos taurus mitogen-activated protein kinase kinase kinase 8
	307	-42.9	TTN	titin
	317	-43.86	F9	Bos taurus coagulation factor IX (F9)
	329	-43.52	CCDC176	coiled-coil domain containing 176
bta-novel-miR-11	172	-20.38	ZMYM6	Bos taurus zinc finger, MYM-type 6
	173	-25.23	C1orf110	chromosome 1 open reading frame 110
	173	-20.07	RTN1	Reticulon
	174	-22.6	PDGFRL	Bos taurus platelet-derived growth factor receptor-like (PDGFRL)
	175	-21.36	C1orf101	chromosome 1 open reading frame 101
	175	-20.13	C16H1orf227	Bos taurus chromosome 16 open reading frame, human C1orf227
	179	-20.58	MOK	MOK protein kinase
	180	-22.75	JAKMIP1	Bos taurus janus kinase and microtubule interacting protein 1
	322	-41.44	TAF1D	Bos taurus TATA box binding protein (TBP)-associated factor, RNA polymerase I, D
bta-novel-miR-2	329	-50.64	ANKRD24	ankyrin repeat domain 24
	330	-50.15	FLNB	filamin B, beta
	344	-48.16	UTRN1	utrophin
bta-novel-miR-4	172	-29.16	SLC7A6-202	Bos taurus solute carrier family 7 (amino acid transporter light chain, y+L system), member 6 (SLC7A6)
bta-novel-miR-6	173	-26.07	NXPE3	NXPE family member 3
	173	-24.45	PFDN4	prefoldin subunit 4
	174	-27.59	SLIT2	slit homolog 2 (Drosophila)
	175	-24.11	ANO4	Bos taurus anoctamin 4 (ANO4)
bta-novel-miR-9	172	-20.05	HIF3A-201	Bos taurus hypoxia inducible factor 3, alpha subunit
	173	-23.59	DGKE	diacylglycerol kinase, epsilon
	174	-20.88	CD46	CD46 molecule, complement regulatory protein
	175	-20.75	LRRC63	leucine rich repeat containing 63
	177	-21.97	PHKA2	phosphorylase kinase, alpha 2
	178	-23.1	DPY19L2	dpy-19-like 2 (C. elegans)
	179	-27.72	CCNG2	cyclin G2

Table S4. Target genes for the 6 differentially expressed novel miRNAs in testis from cattleyak (CY) versus yak (YK) are shown, using target mining software of miRanda. MiRNAs with > 300 predicted targets in genome were excluded, whereas target genes with ⩾ 172 total score were shown.

MiRNA ID	Stem-loop RT primer (5′−3′)	MiRNA specific forward primer (5′−3′)	Universal reverse primer (5′−3′)
bta-miR-449a	gtgatccagtcggtccgaggtcagattccgactggatcacACCAGCTA	gcgcgTGGCAGTGTATTGTT	gtcggtccgaggtcag
bta-miR-449b	gtgatccagtcggtccgaggtcagattccgactggatcacGCCAGCTA	gcgcgAGGCAGTGTATTGTT	gtcggtccgaggtcag
bta-miR-19b	gtgatccagtcggtccgaggtcagattccgactggatcacTCAGTTTTG	gcgcTGTGCAAATCCATGC	gtcggtccgaggtcag
bta-miR-184	gtgatccagtcggtccgaggtcagattccgactggatcacACCCTTATC	gcgTGGACGGAGAACTGA	gtcggtccgaggtcag
bta-miR-378	gtgatccagtcggtccgaggtcagattccgactggatcacGCCTTCTG	gcgACTGGACTTGGAGTC	gtcggtccgaggtcag
bta-miR-135	gtgatccagtcggtccgaggtcagattccgactggatcacTCACATAGG	gcgcgcTATGGCTTTTTATTCC	gtcggtccgaggtcag
bta-novel-miR-10	gtgatccagtcggtccgaggtcagattccgactggatcacTCCACTCT	gcgcgcTGATTGGTACTTCTT	gtcggtccgaggtcag
bta-novel-miR-11	gtgatccagtcggtccgaggtcagattccgactggatcacGATTTATAC	gcgcgTTCTCTTCAGATCGT	gtcggtccgaggtcag

Table S9. Stem-loop RT-PCR primers for real-time quantification of selected differentially expressed miRNAs in testis between cattleyak and yak.

Gene symbol	Forward primer (5′−3′)	Reverse primer (5′−3′)	PCR product (bp)
E2F5	acctatccacgtgctgctta	gcttcgttcagagacaggtg	178
CCNT2	tgatgaaggcagtgggaaga	gctattccgtcagcactgtg	171
NCOR2	gaccgaaagctgacatccac	ggattcctcggggtatggag	181
MAPK1IP1L	ttcctgagcttccaagacca	tggccctggagatggatatg	152
ZNF226	ccttcagtcccaccagagag	tgaagacttgaaggccgact	174
MAP3K8	cctcagtcagatacgggacc	tgtaggtcagcttccaaggg	189
JAKMIP1	tcagatcgagttccagcagg	tccttggtcttctcctcgtg	150
β-actin	aagttctacagtgtggccga	gactggccccttctccttag	150
RPS18	cgccgccatgtctctagtaa	aggtcgatgtctgctttcct	162

Table S10. Primer sequences for real-time quantification of the target genes of selected differentially expressed miRNAs.
